# Xylose Acetals ‐ a New Class of Sustainable Solvents and Their Application in Enzymatic Polycondensation

**DOI:** 10.1002/cssc.202401877

**Published:** 2024-11-20

**Authors:** Anastasia O. Komarova, Cicely M. Warne, Hugo Pétremand, Laura König‐Mattern, Johannes Stöckelmaier, Chris Oostenbrink, Georg M. Guebitz, Jeremy Luterbacher, Alessandro Pellis

**Affiliations:** ^1^ Laboratory of Sustainable and Catalytic Processing Institute of Chemical Sciences and Engineering École Polytechnique Fédérale de Lausanne (EPFL) Station 6 1015 Lausanne Switzerland; ^2^ acib GmbH Konrad-Lorenz-Strasse 20 3430 Tulln an der Donau Austria; ^3^ Institute of Environmental Biotechnology Department of Agrobiotechnology IFA-Tulln University of Natural Resources and Life Sciences Vienna Konrad-Lorenz-Strasse 20 3430 Tulln an der Donau Austria; ^4^ Max Planck Institute for Dynamics of Complex Technical Systems Process Systems Engineering (PSE) Sandtorstraße 1 D-39106 Magdeburg Germany; ^5^ Institute of Molecular Modeling and Simulation (MMS) University of Natural Resources and Life Sciences Vienna Muthgasse 18 1190 Vienna Austria; ^6^ Universitá di Genova Dipartimento di Chimica e Chimica Industriale via Dodecaneso 31 16146 Genova (GE) Italy

**Keywords:** Biocatalysis, Biomass valorisation, Green chemistry, Polymers, Solvent, Xylose

## Abstract

The use of organic solvents in academic research and industry applications is facing increasing regulatory pressure due to environmental and health concerns. Consequently, there is a growing demand for sustainable solvents, particularly in the enzymatic synthesis and processing of polyesters. Biocatalysts offer a sustainable method for producing these materials; however, achieving high molecular weights often necessitates use of solvents. In this work, we introduce a new class of alternative aprotic solvents with medium polarity produced directly from agricultural waste biomass in up to 83 mol % yield (on xylan basis). The new solvents have a largely unmodified xylose core and acetal functionality, yet they show no peroxide formation and provide reduced flammability risk. We also demonstrate their successful application in enzymatic polycondensation reactions with *Candida antarctica* lipase B (CaLB). In particular, the solvent dibutylxylose (DBX) outperformed the hazardous solvent diphenyl ether and facilitated polycondensation of the lignin‐derived diester pyridine‐2,4‐dicarboxylate, yielding polyesters with a M_n_ of >15 kDa. Computational modelling studies provided further insight into the molecular structure and dynamics of CaLB in the presence of new solvents. Lastly, up to 98 wt % of the new xylose acetals were successfully recovered and recycled, further contributing to the sustainability of the overall process.

## Graphical abstract

Novel organic solvents with an unmodified carbohydrate core were synthesised both from D‐xylose and directly from biomass and were successfully used as reaction media for lipase biocatalysts to synthesise aliphatic and aromatic polyesters.

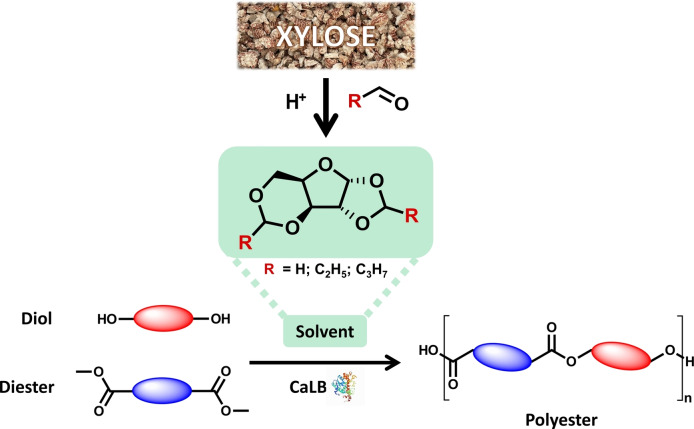

## Introduction

Organic solvents are a fundamental part of chemistry with applications in both formulation and synthesis. A solvent typically accounts for more than 50 % of the total mass of all ingredients in the reaction medium, making it one of the main contributors to the overall process performance.^[1]^ Yet, the use of organic media is far from ideal in terms of Environment, Health, and Safety (EHS) principles. According to a recent survey of publications in three representative chemistry journals, the majority of solvents regularly used in reactions include problematic toxic solvents such as dichloromethane (DCM), tetrahydrofuran (THF), methanol, dimethylformamide (DMF), toluene among others.^[2]^ A similar analysis by Syngenta in 2018–2019 revealed that the aforementioned solvents were used in about 60 % of all reactions performed.^[3]^ The hazards posed by these solvents, from carcinogenicity to flammability, as well as their non‐sustainable production from fossil sources, raise significant concerns and have led to tightening legislation. Particularly concerning are aprotic solvents, especially polar aprotic solvents and ethers as reported in various solvent selection guides[^4^, ^5^, ^6^, ^7^] and in the European REACH regulation.[^8^, ^9^] The main concern associated with the use of polar aprotic solvents is their reprotoxicity, while most ethers present a serious safety risk due to their ability to form explosive peroxides during use and storage. In this regard, the search for greener and safer alternative solvents, preferably produced from renewable sources is one of the key green chemistry research areas.

A common approach for developing alternative renewable solvents revolves around a carbohydrate platform. Carbohydrates are abundant in nature and can be derived from various biomass sources, including agricultural non‐edible by‐products, forestry residues, food scraps, and industrial sugar‐rich waste streams. However, due to the abundance of free hydroxyl groups in natural carbohydrates, the production of aprotic solvents from biomass is challenging and often requires expensive prior transformations via dehydration and hydrogenation chemistry. One such example is 2‐methyltetrahydrofuran (2‐MeTHF), a cyclic ether produced by hydrogenation of carbohydrate‐derived furfural or levulinic acid.^[10]^ Bio‐based 2‐MeTHF is a non‐mutagenic and non‐genotoxic solvent^[11]^ that offers a lower carbon footprint^[12]^ when compared to traditional tetrahydrofuran (THF). However, 2‐MeTHF is still considered problematic due to its high flammability, volatility, corrosiveness, and elevated cost. The latter factor also significantly limits the use of γ‐valerolactone (GVL) ‐ another solvent that can be produced by hydrogenation of levulinic acid^[13]^ or furfural.^[14]^ Hydrogenation of levoglucosenone (LGO), a platform molecule produced from cellulose, gives another alternative solvent Cyrene, which is also commercially available. Cyrene and its derivatives (Cygnets) have comparable performance to reprotoxic NMP in cross‐coupling and fluorination reactions as well as polymer chemistry applications,^[15]^ although they suffer from low stability in the presence of acids and oxidizers.^[16]^ Tetrahydropyran (THP) recently appeared as a new potential substitute for THF but its production from furfural‐derived 3,4‐dihydropyran (DHP) still requires several hydrogenation and dehydration steps.^[17]^ Another cyclic ether 2,5‐dimethyltetrahydrofuran (DMTHF) can be produced from various bio‐based molecules by hydrogenation[^18^, ^19^] or from 2,5‐hexanediol via an innovative acid‐free dehydrative cyclisation process.^[20]^ However, the peroxide formation rate of DMTHF was only slightly slower than that of 2‐MeTHF, suggesting similar safety issues. Two recently introduced non‐polar cyclic ethers without a proton at the alpha‐position to the oxygen, a structural element responsible for peroxide formation risks, are 2,2,5,5‐tetramethyloxolane (TMO) and 2,5‐diethyl‐2,5‐dimethyloxolane (DEDMO). Both solvents can be produced from biomass via multi‐step hydrogenation‐dehydration reactions in the presence of Pd‐based or zeolite catalysts.[^21^, ^22^] Dimethylimidazolidinone (DMI) and 1,3,4‐trimethylimidazolidin‐2‐one (TMI) are interesting alternative candidates to polar aprotic solvents known since the 1990s, but were only recently produced from renewable cellulose in one‐pot catalytic process using Ru/C.^[23]^ Overall, these examples illustrate the efforts to respond to industrial demands and create bio‐based aprotic solvents with attractive EHS properties produced by simple, high‐yield routes from renewable sources.

We have previously introduced a novel polar aprotic solvent and platform molecule, diformylxylose (DFX), that can be produced in a single step from the xylan fraction of lignocellulosic biomass via aldehyde‐assisted fractionation (AAF).[^24^, ^25^] In this approach, we use inexpensive bulk chemical formaldehyde and acid catalyst to stabilize reactive hydroxyl groups in xylose in the acetalised form. This strategy not only isolates xylose directly in an aprotic form but also achieves high yields by minimising sugar degradation. DFX has proved to be a non‐mutagenic solvent with demonstrated efficacy in hydrogenation, alkylation, and cross‐coupling reactions.^[16]^ In recent work, we showed that DFX is inherently biodegradable and scaled up the production of DFX from corn cobs to a multi‐kg scale in a sustainable one‐pot process.^[28]^ By adapting the same process, in this work we expand the portfolio of xylose‐based solvents by introducing various short‐chain aliphatic aldehydes into the xylose core. The resulting xylose acetals are interesting candidates for biocatalysis and, specifically, for polyester synthesis with *Candida antarctica* lipase B (CaLB) due to their properties discussed in this work.

Polyesters are a class of materials with well‐known environmental issues yet are essential for many applications such as food packaging,^[29]^ clothing,^[30]^ and biomedical applications.^[31]^ Conventional synthesis of polyesters typically requires the use of metal catalysts at elevated temperatures.^[32]^ Although many industrial processes for producing polyesters are solvent‐free, the use of toxic, non‐renewable metal catalysts and the need for energy‐intensive conditions reduces the overall sustainability. Alternatively, lipase biocatalysis offers an efficient and inherently green synthesis of both aliphatic and aromatic polyesters.[^33^, ^34^] However, use of a biocatalyst at mild temperatures generally results in increased viscosity and eventual solidification of the reaction mixture as the polymer molecular weight increases. In such cases, the use of a solvent to solubilize the polymer chains is necessary to achieve high molecular weights, as temperatures cannot be increased without denaturing the enzyme. The traditional solvent choice for polycondensation reactions is environmentally hazardous diphenyl ether (DPE).^[35]^ Ether solvents, in general, have proven to be effective in these reactions due to their unreactivity towards lipases, and their medium polarity which is especially favourable to CaLB catalysis.^[36]^ Unfortunately, many traditional ether solvents are toxic, petroleum‐based, and can form explosive peroxides. In search for greener alternatives, various ionic liquids and deep eutectic solvents (DES) have been explored as media and several of them have shown compatibility with enzymes.[^15^, ^37^, ^38^, ^39^] However, there are significant concerns over the toxicity of both classes of solvents.[^40^, ^41^] In addition, lipases recognize many DES components as substrates,^[42]^ hindering the synthesis. In this work, we demonstrate the application of novel bio‐based, non‐peroxide forming xylose acetals as solvents in CaLB‐catalysed polycondensation reactions for the synthesis of aliphatic and aromatic bio‐based polyesters, as well as the successful recovery and recycling of these new solvents.

## Results and Discussion

### Production of Xylose Acetals from D‐Xylose and Xylan‐Rich Biomass

**Figure 1:**
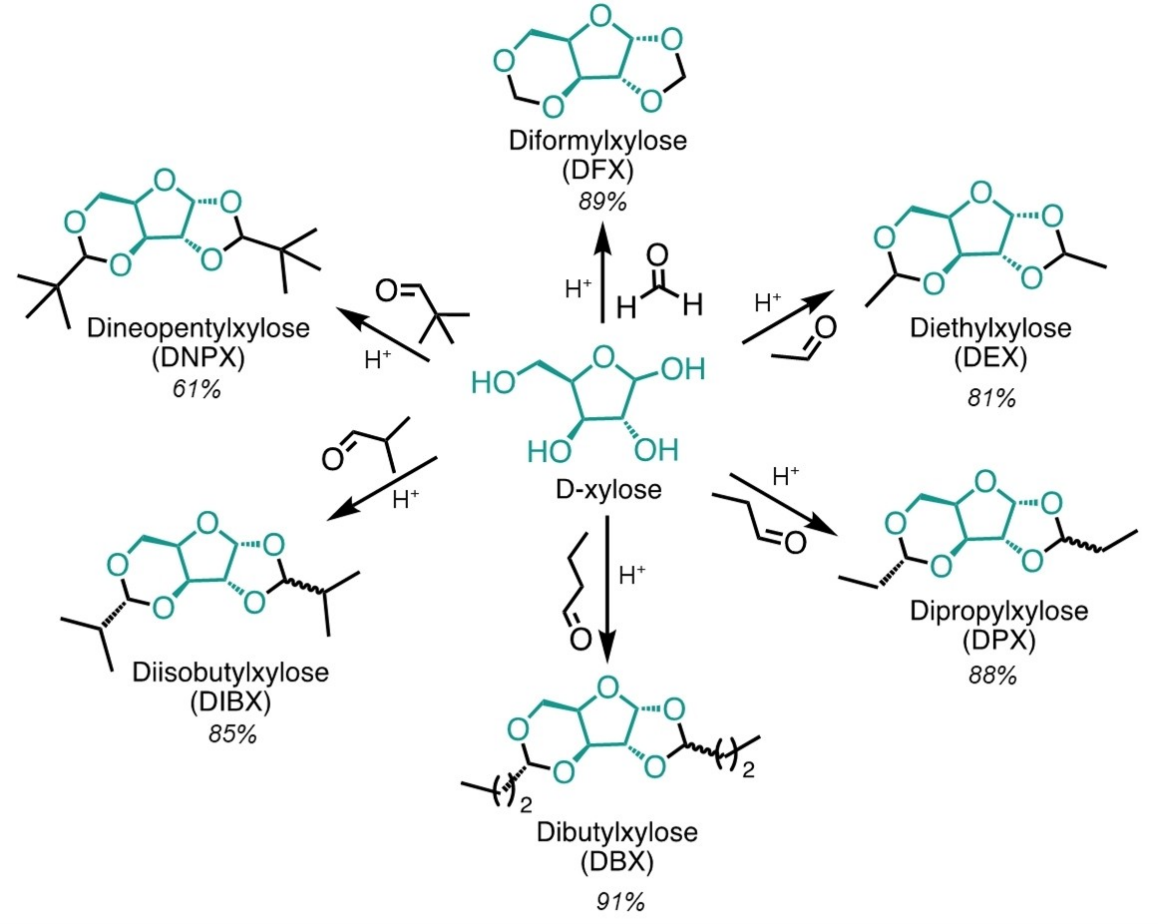
DFX and other xylose acetals synthesised from D‐xylose using the corresponding aldehyde and acid catalyst. The reaction yields in mol % of xylose are provided in italics. The full stereochemistry was not investigated further for DEX and DNPX and so is not shown here.

D‐xylose, purified or as part of the xylan‐rich biomass, can be acetalised using formaldehyde and mineral acid to produce diformylxylose (or DFX) in a simple and scalable one‐pot process in >80 % yield.^[28]^ The use of propionaldehyde in a similar process gives dipropylxylose (DPX)^[27]^ that has never been explored as a solvent. Here, we explore this and four new acetal‐stabilised molecules synthesised from pure D‐xylose for the first time: diethylxylose (DEX), dibutylxylose (DBX), diisobutylxylose (DIBX), and dineopentylxylose (DNPX) (Figure 1). We synthesised these molecules in the bio‐based solvent 2‐MeTHF with a catalytic amount of H_2_SO_4_ in mild conditions (60–80 °C for 1–3 h), which afforded products in over 80 mol % yield at up to 100 mol % of xylose conversion (see Supplementary Information (SI) Tables S1 and S2 for details). We observed a lower xylose conversion (81 mol %) only when synthetizing DNPX, which reduced the reaction yield (61 mol %) likely due to the steric hindrance of the quaternary carbon. After neutralisation and removal of the reaction solvent, the products readily crystallised into colourless crystals with over 95 % purity (full structure characterisation of the synthesised molecules can be found in SI, Figures S1–S5).

The introduction of these aliphatic functional groups onto the xylose core generated two new asymmetric carbon atoms, resulting in the formation of a mixture of diastereomers. We detected the presence of two diastereomers for DPX, DBX and DIBX by gas chromatography (GC), while for DEX and DNPX we observed only one peak. Previous research of our group on DPX showed that both diastereomers form in the 5‐member ring as revealed by ^1^H‐nuclear magnetic resonance (NMR) spectroscopy.^[27]^ NMR analysis of DBX and DIBX showed similar signal patterns to those of DPX (see SI, Figures S2–S4). Additionally, X‐ray analysis of a single crystal (see SI, Table S15 for crystallography data) revealed that the diastereomer with over 75 % abundance has an S configuration at the 5‐membered ring acetal carbon. However, the diastereomer ratio is influenced by reaction conditions, with higher temperatures and longer reaction times favouring the alternate R diastereomer. These results align with the general conception proposed by Clode^[43]^ that an acid‐catalysed acetalisation reaction first produces the “kinetic product” that forms faster (the S diastereomer in this case), while R diastereomer forms at the later phase of the reaction as a the thermodynamically stable product.

**Figure 2:**
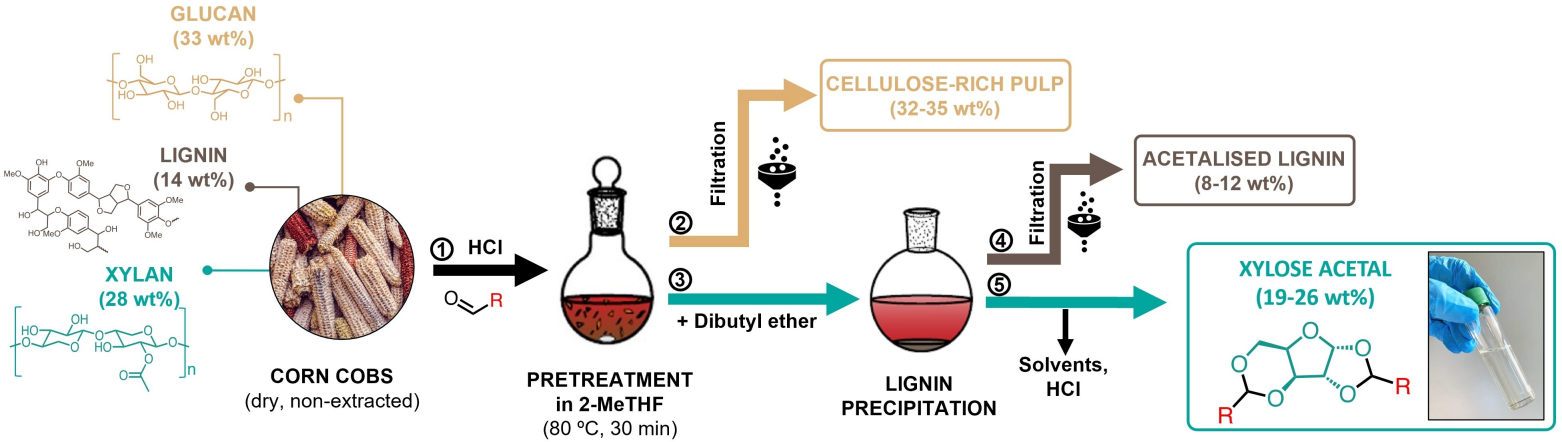
Schematic overview of the process for isolating four xylose acetals (DFX, DPX, DBX, DIBX, see full structures in Figure 1) from corn cobs. Weight percentages are based dry and non‐extracted biomass. The values for xylose acetals and acetalised lignin are corrected for the mass of added water and corresponding aldehyde to represent the fraction of the original biomass that ends up in the final product.

We also isolated xylose acetals directly from corn cobs (see SI, Section S3.2 for method details) ‐ a xylan‐rich (26 wt % of xylan, see SI, Table S4 for full compositional analysis) agricultural waste available in large quantities worldwide. Organosolv pretreatment of biomass in 2‐MeTHF in the presence of HCl and the corresponding aldehyde under mild condition (80 °C, 30 min) was followed by fractionation to isolate xylose acetals as well as the two other fractions ‐ cellulose‐rich pulp and uncondensed acetal‐stabilised lignin (Figure 2). The fractionation method used in this work was adapted from the original procedure developed by our group for multi‐kg scale production of DFX from corn cobs.^[28]^ This previously reported method was shown to be scalable, cost‐competitive, with low environmental impact and a net negative global warming potential at the factory gate.^[28]^ Briefly, after separating the non‐soluble pulp from the pretreated mixture by filtration, we added dibutyl ether to the mixture to precipitate lignin and then separated it by filtration as well. To isolate the xylose acetal, we removed solvents and HCl from the mixture by evaporation. The resulting oil was crystallised and washed with ethanol to obtain the pure product. We achieved over 90 mol % reaction yield of xylose acetals (on a xylan basis) after pretreatment. After performing full fractionation and purification, we isolated target products (DFX, DPX, DBX, and DIBX) in >70 mol % on a xylan basis (the yields for each molecule are provided in SI, Table S3).

**Figure 3:**
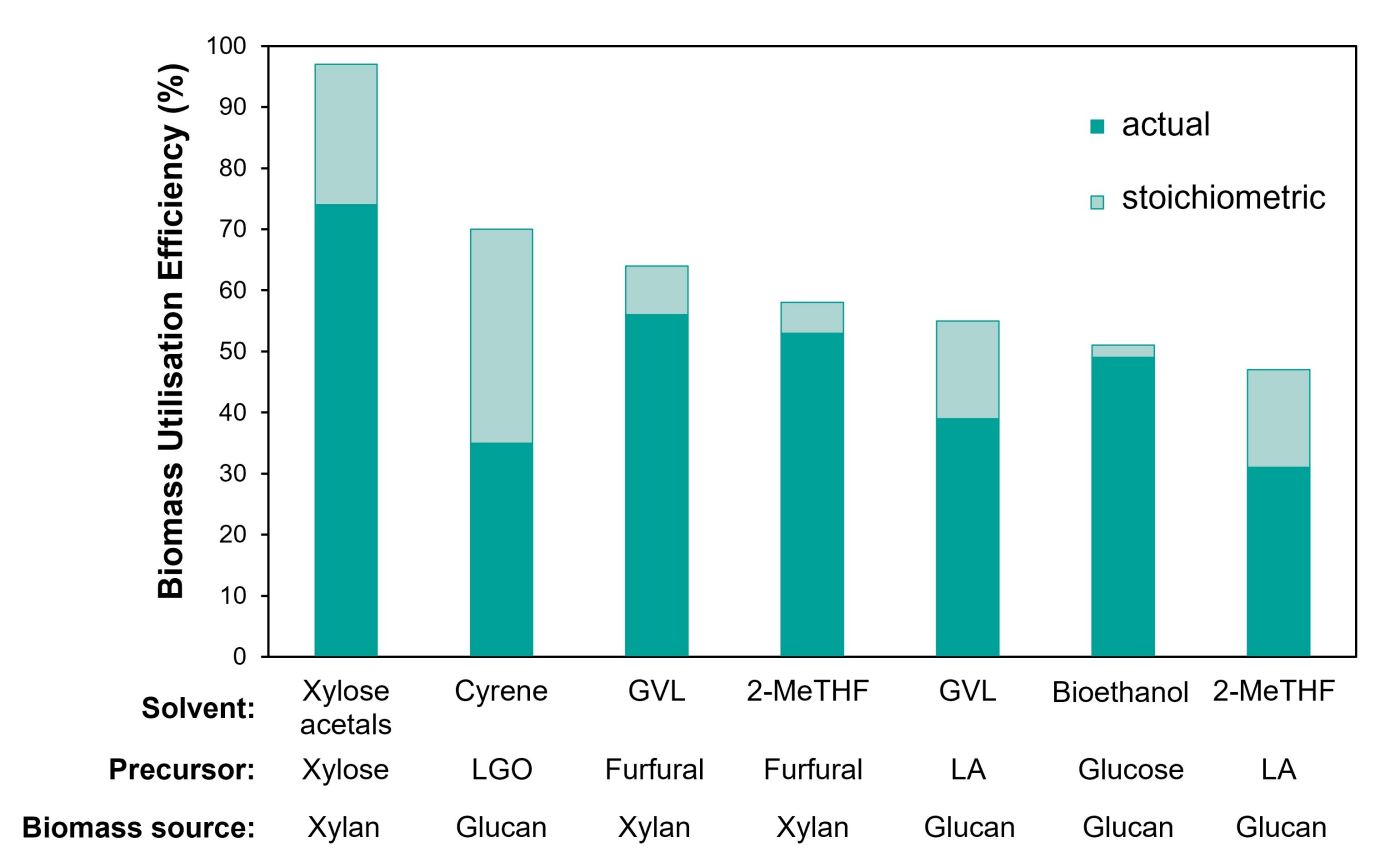
Biomass utilisation efficiency (stoichiometric in light turquoise colour and actual in dark turquoise colour) for xylose acetals produced by the aldehyde‐assisted fractionation compared to other selected solvents produced from their precursors derived from natural sugars (glucan or xylan) or produced by fermentation (bioethanol). The value for xylose acetals was averaged for DFX, DPX, DBX, and DIBX. Cyrene, GVL, 2‐MeTHF, and bioethanol values were calculated assuming 100 % efficiency of xylan or glucan separation from biomass and 100 % yield of xylose from xylan and glucose from glucan. The detailed calculation is provided in SI, Section S3.3.

In xylose acetals, 97 % of biogenic xylose atoms are preserved, as they originate from the xylose core that remains intact within the solvent structure. Indeed, only four hydrogen atoms in xylose are substituted to produce the final product, leading to a high stoichiometric biomass utilisation efficiency, a metric that shows how much of the initial biomass theoretically ends up in the final product (Figure 3).^[44]^ The actual biomass utilisation efficiency based on the highest isolated yield (70–83 mol % on xylan basis) accounts for approximately 74 % on average for DFX, DPX, DBX, and DIBX, produced by the described method (see SI for detailed calculation). These results demonstrate that xylose acetals produced by the AAF have a very interesting advantage in terms of sustainability and economic viability over other commercialised bio‐based solvents (Figure 3). Further enhancing the sustainability of solvent production could involve using aldehydes produced from renewable sources through chemical or biological methods. For instance, formaldehyde from biomethanol, propionaldehyde from bio‐derived 1,2‐propylene glycol,^[45]^ butyraldehyde from glucose,^[46]^ and isobutyraldehyde from CO_2_.^[47]^ However, bio‐based versions of these aldehydes are still under development and are not yet commercially available.

### Physical Properties of Xylose Acetals

Diformylxylose (DFX) emerged as a promising bio‐based alternative to traditional polar aprotic solvents in our past work, despite having a relatively high melting point of 48 °C.^[16]^ The introduction of flexible aliphatic chains in the xylose core in this work lowered melting points of DPX, DBX, and DIBX compared to DFX (Table 1), although they slightly varied for each compound depending on the ratio of diastereomers formed (see SI, Table S6 for melting points measured across various diastereomer ratios). Notably, the isolated pure DPX with S/R ratio of 5.5 : 1, DBX with S/R ratio of 6 : 1, and DIBX with S/R ratio of 6.5 : 1 are liquids at room temperature range (23–25 °C). This fact extends their applicability over DFX by providing greater flexibility in reaction temperature. At the same time, the melting points of DEX and DNPX are significantly higher than that of DFX (60 and 120 °C, respectively). Consequently, we excluded DEX and DNPX from further investigation as potential solvents due to limitations associated with elevated melting points.

**Table 1:** Physical and solvation properties of xylose acetals and selected traditional solvents.

Properties	Xylose acetals				Polar aprotic solvents		Traditional ethers		
Name	DFX	DPX	DBX	DIBX	DMF	NMP	THF	1,4‐Dioxane	DPE
Structure	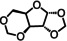	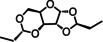	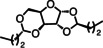	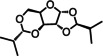	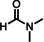	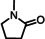	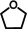	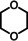	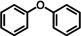
Source/precursor	Biomass/xylose	Biomass/ xylose	Biomass/ xylose	Biomass/ xylose	Petroleum/dimethylamine	Petroleum/2‐pyrrolidone	Petroleum/1,4‐butanediol	Petroleum/ethylene oxide	Petroleum/chlorobenzene
Molecular weight, g/mol	174.15	230.26	258.31	258.31	73.09	99.13	72.11	88.1	170.21
Melting point, °C	48	28±5^[a]^	27±3^[a]^	30±5^[a]^	−78	−24	−108	12	28±2
Boiling point (1 atm), °C Boiling point (0.008 atm), °C	237 96	238 97	235 95	235 95	153 –	202 –	66 –	101 –	259 –
Density (25 °C), g/ml	1.35^[b]^	1.17^[b]^	1.15^[b]^	1.11^[b]^	0.94	1.03	0.89	1.03	1.07
Solubility in water (25 °C), wt %	13	1.8	<0.10	<0.10	Miscible	Miscible	Miscible	Miscible	<0.10
Flash point, °C	138	138	156	144	58.0	91.0	‐14.0	12.0	115
Peroxide formation, mmol/kg	None^[c]^	None^[c]^	None^[c]^	None^[c]^	None	None	>10^[d]^	4^[d]^	N/A
δD/MPa^0.5^	17.9	16.4	16.3	16.2	17.4	18.0	16.8	17.3	19.4
δP/MPa^0.5^	9.0	6.1	5.2	4.9	13.7	12.3	5.7	4.3	3.4
δH/MPa^0.5^	7.6	4.5	3.8	3.2	11.1	7.2	8.0	8.4	4.0
α	0.00	0.00	0.00	0.00	0.00	0.00	0.00	0.00	0.00
β	0.82	0.63	0.56	0.51	0.69	0.75	0.55	0.37	0.13
π*	0.92	0.68	0.65	0.63	0.88	0.90	0.58	0.55	0.66
Nile Red λ_max_, nm	543	536	534	535	541	541	528	516	N/A

[a] for the mixture with ≥75 % of the S diastereomer, [b] at 50 °C, [c] after 22 months of storage, [d] after 3 months of storage without stabiliser.^[55]^ N/A ‐ not available, DFX ‐ diformylxylose, DPX ‐ dipropylxylose, DBX ‐ dibutylxylose, DIBX ‐ diisobutylxylose, DMF ‐ dimethylformamide, NMP ‐ N‐methyl‐2‐pyrrolidone, THF ‐ tetrahydrofuran, DPE ‐ diphenyl ether.

The boiling points of all synthesised xylose acetals are higher than 230 °C (Table 1), putting them in line with other high‐boiling industrial solvents such as sulfolane (285 °C), dipropylene glycol (231 °C) and diethyl phthalate (295 °C). The advantage of a high boiling point is reduced risk of inhalation or exposure due to released vapours at normal operating temperatures. On the other hand, high‐boiling solvents often require separation and recovery techniques that differ from traditional distillation. Several common methods, including product precipitation,^[48]^ liquid‐liquid extraction,[^49^, ^50^] adsorption,^[51]^ and facilitated distillation,^[52]^ have been successfully employed for solvents with boiling points higher than 200 °C, especially in industrial settings.

The notably high flash points (*fp*) of all xylose acetals (≥138 °C) put them in the non‐flammable solvent category and provide reduced safety risks in processes involving heat. This feature also makes the handling, storage, and transporting of xylose acetals safer and easier compared to traditional flammable solvents such as 2‐MeTHF (*fp* −10 °C), THF (*fp* −14 °C), 1,4‐dioxane (*fp* 12 °C), diethyl ether (*fp* −45 °C). The use of non‐flammable solvents is especially desired in industry as it ensures compliance with safety regulations and leads to cost savings by eliminating the need for specialised equipment and extra safety measures.

DFX exhibits limited solubility in water (13 wt % at 25 °C) similar to 2‐MeTHF (15 wt % at 25 °C). In contrast, DPX, DBX, and DIBX are insoluble in water, which opens new opportunities for their recovery such as extraction from the aqueous to the organic phase and/or precipitation from a solution triggered by the addition of water. To further explore the feasibility of these separation techniques for xylose acetals, we determined the miscibility of DFX and DBX with 16 common organic solvents (see SI, Table S9). DFX was found to be immiscible with low‐polarity solvents, including cyclohexane, dibutyl ether, and hexane due to its own high polarity, whereas DBX is only immiscible with water. Similar miscibility behaviour is expected for DPX and DIBX due to their structural similarity with DBX.

Xylose acetals demonstrated remarkable resistance to peroxide formation after a minimum of 22 months of storage in non‐sealed containers, with DFX confirmed stable for up to 3 years, as determined by the iodometric titration method (see SI, Section S3.8 and Table S10 for details). Although acetals, in general, are less prone to peroxide formation than ethers, the presence of alpha protons in the acetal structure also leads to the initiation of free‐radical reactions.^[53]^ For example, acetaldehyde diethyl acetal (or 1,1‐diethoxyethane) is classified as a level 2 risk (on a scale of 1–3) for peroxide formation and requires extra caution during evaporation, distillation, and storage longer than 3 months. In contrast, xylose acetals showed exceptional stability against peroxide formation that can be attributed to several factors. First, the cyclic structure with envelope conformation, as revealed by X‐ray analysis and supported by the literature,^[54]^ introduces steric hindrance, limiting accessibility of reactive sites. The C−H⋯O hydrogen bonds observed in DFX between adjacent molecules form a stabilising three‐dimensional network that reduces sensitivity of the molecule to the attack of reactive radicals. In DPX, DBX, and DIBX, bulky alkyl chains can hinder their interaction with reactive species. Lastly, stable molecular packing in crystals of xylose acetals that have relatively high melting point (and DFX is solid at room temperature) further contributes to the slowed down diffusion of reactive species. In this regard, xylose acetals could be safe alternatives to traditional peroxide‐forming acetals and ethers.

A summary of the limitations and advantages of xylose acetals in terms of practical use, particularly for large‐scale industrial settings, along with safety and environmental considerations, is provided in the SI, Section S4.

### Solvation Properties of Xylose Acetals

**Figure 4:**
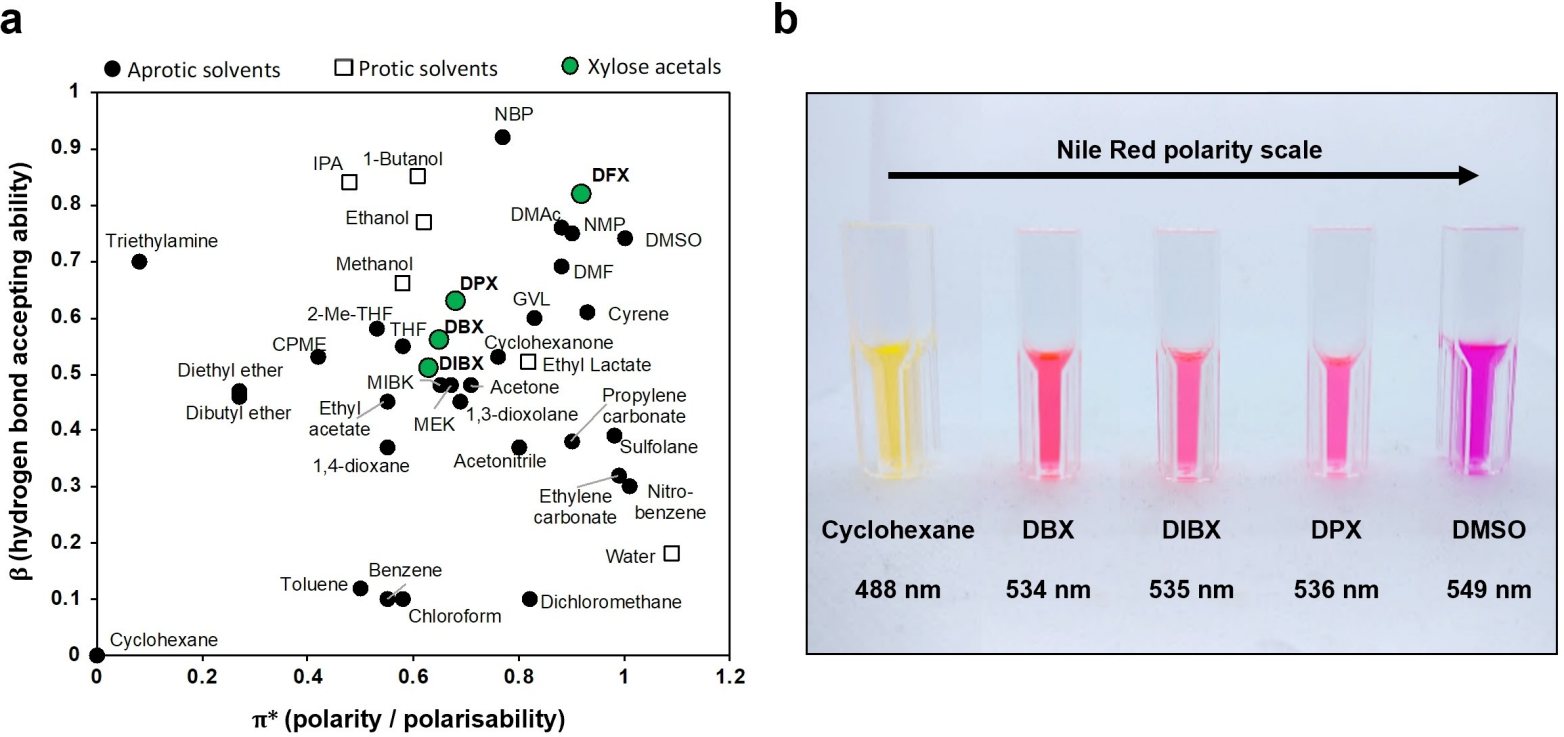
**(a)** Kamlet‐Abboud‐Taft solvent map showing aprotic solvents as black circles, protic solvent as white squares, and xylose acetals as green circles. **(b)** Picture of the mixtures with Nile Red dye dissolved in xylose acetals, cyclohexane, and DMSO in a concentration of 24 μM. The values in nm indicate the wavelength of the maximum absorbance. The UV‐Vis spectra are provided in SI, Figure S7.

To evaluate the solvation properties of xylose acetals we experimentally measured three Kamlet‐Abboud‐Taft (KAT) solvatochromic parameters: α ‐ hydrogen bond donating ability, β ‐ hydrogen bond accepting ability, and π* ‐ polarity and polarisability of solvent (Table 1).^[56]^ The KAT parameters of xylose acetals were obtained from the UV‐Vis spectra of a set of solvatochromic dyes dissolved in the solvents (see SI, Section S3.4 and Figure S6 for details). The obtained π* values for DPX, DBX, and DIBX (0.68, 0.65, 0.63) characterise them as medium‐polarity solvents. The β values for these solvents also fall within the middle range (0.63, 0.56, 0.51). COSMO‐RS modelling confirmed that these xylose acetals are hydrogen bond acceptors due to the high electronegativity of the oxygen atoms in the xylose core (see SI, Figure S15). Plotting measured π* against β illustrates that DPX, DBX, and DIBX occupy the part of a solvent map populated by solvents such as methyl isobutyl ketone (MIBK), methyl ethyl ketone (MEK), THF, acetone, and 1,3‐dioxolane (Figure 4, a). In line with this observation, DPX, DBX, and DIBX demonstrated similar performance to MEK and cyclic ethers in the Menshutkin alkylation reaction, where the reaction rate is nearly linearly proportional to the π* parameter (see SI, Figure S20). Among molecules recently explored as solvents, 1,3‐dioxolan‐4‐ones (DOXs)^[57]^ and tetrahydropyran (THP)^[17]^ have the closest match with xylose acetals in the scope of the KAT model.

Importantly, the π* parameter in KAT model determines a solvent‘s polarity combined with its polarisability, with the latter being influenced by molecular weight. Since xylose acetals have relatively high molecular weight, the contribution from their polarisability might overtake the effect of their polarity when evaluating and comparing π* values. To address this concern, we also used the Nile Red metric to assess solely these molecules’ polarity.^[58]^ The maximum absorbance of Nile Red dye dissolved in a solvent of interest depends on both polarity and acidity, while the acidity is zero for aprotic solvents like xylose acetals. The results show that DPX, DBX, and DIBX are indeed medium‐polarity solvents (534–536 nm) that are slightly more polar than THF (528 nm) and acetone (534 nm) but still less polar than DMF (541 nm), DFX (543 nm), and DMSO (549 nm) (Figure 4, b).

KAT parameters are often used to correlate solvent properties with reaction rates, equilibria, and other dynamic chemical phenomena. In addition, Hansen Solubility Parameters (HSP) provide a method for evaluating the inherent solvency power from a different perspective. HSP theory breaks down the solvent's cohesive energy density into three components: dispersion forces (δ_D_ term), polar forces (δ_P_ term), and hydrogen‐bonding forces (δ_H_ term).^[59]^ We predicted the corresponding HSP parameters based on the structures of xylose acetals using HSPiP software. According to the determined values (see SI, Table S5), the closest matching solvents for DPX, DBX, and DIBX in Hansen space are methyl isoamyl ketone (MIAK, which is a solvent used in various resins,^[60]^ coatings,^[61]^ adhesives,^[62]^ etc.), tributyl phosphate (TBP, which is used as an industrial solvent and an extractant for metal ions^[63]^), CPME, methyl oleate, and MIBK. These results suggest that xylose acetals could be used in similar applications to the aforementioned solvents while offering enhanced sustainability.

### Xylose Acetals in the Sustainable Synthesis of Polyesters with CaLB

The solvation properties of xylose acetals determined in the above section such as relatively low β and π* values combined with their high molecular weight make them attractive candidates for biocatalysis with CaLB. These solvent characteristics have been shown to positively affect the biocatalytic performance of lipase B, leading to higher rates and yields.[^64^, ^65^] Indeed, all three terms are components of linear free energy relationship (LFER) used to define partition coefficient (*log P*),^[66]^ that has been shown to affect biocatalytic activity.[^36^, ^67^, ^68^]

**Figure 5:**
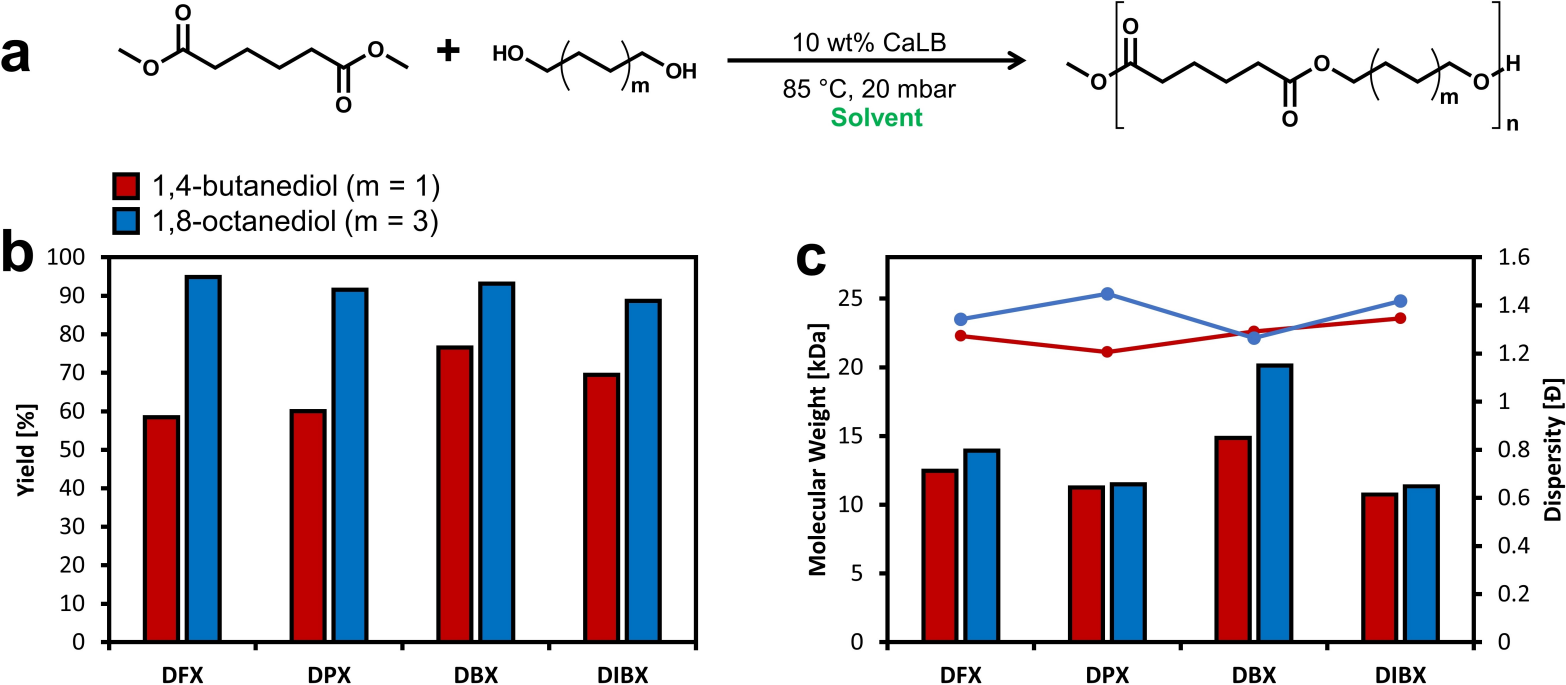
**(a)** Polycondensation reaction scheme of dimethyl adipate with diol catalysed by lipase B from *Candida antarctica* (CaLB). **(b)** Yields of aliphatic polyesters produced in xylose acetals. **(c)** Number average molecular weights (Mn) of the produced polyesters shown as bars and dispersity shown as lines. Data for BDO are coloured in red and for ODO in blue.

For the first representative polycondensation reaction in xylose acetals with CaLB, we used the following aliphatic monomers: dimethyl adipate (DMA) paired with either 1,4‐butanediol (BDO) or 1,8‐octanediol (ODO) (Figure 5, a). This benchmark reaction is well‐studied and known to proceed efficiently using CaLB.[^37^, ^69^] The reaction proceeded at 85 °C under reduced pressure, and the resulting polymers were recovered by dissolution in 2‐MeTHF followed by precipitation in methanol. Notably, the yield of isolated poly(1,8‐octylene adipate) in all xylose acetals that were tested was consistently over 89 %. Thermal analysis of isolated polyesters showed very little difference in terms of glass transition temperature (*T_g_*) and melting point (*T_m_*) (see SI, Table S14). For the BDO‐based polymer, yields were generally lower, which is in line with previous studies and can be attributed to the enzymes affinity for longer aliphatic substrates.^[35]^ Interestingly, the yields in DBX (77 %) and DIBX (70 %) were considerably higher than those in DPX (60 %) and DFX (58 %), which could be explained by the joint effect of lower HSP and KAT values and higher molar volumes for DBX and DIBX compared to DPX and DFX. Bulkier and less interactive solvents like DBX and DIBX in this context are less likely to diffuse into the enzyme's active site and disrupt the formation of the enzyme‐substrate complex.^[65]^ Additionally, polar media tends to strip water molecules bound to the enzyme, negatively affecting catalysis.^[70]^

The polyesters synthesised in xylose acetals have remarkably low dispersity (Đ) between 1.1 and 1.5, suggesting a more uniform polymer size distribution compared to polymers made in other renewable solvent candidates (*vide infra*). Gel permeation chromatography (GPC) analysis further revealed that in DFX dispersity first rapidly decreases and then stabilizes at 1.3 within 2 hours of the reaction, accompanied by a constant gradual increase in M_n_ (see SI, Figure S8). Polycondensation of the same monomers in Cyrene and Cygnets under similar conditions gave a Đ value between 1.5 and 3.0.^[71]^ Đ values of the same polyesters synthesised in the benchmark solvent DPE fell within a range of 1.1–1.5,^[38]^ indicating the potential of xylose acetals to replace hazardous DPE in this application without sacrificing its benefits. Past work on the polycondensation between dicarboxylic acids and diols has shown that values of Đ below 1.5 are typically obtained in solvent‐free systems.^[35]^ In contrast, when using organic solvent, the dispersity is usually higher and can be explained by the interchain transesterification by CaLB.^[68]^ In xylose acetals, the chain growth reaction appears to be favoured over interchain transesterification, likely due to the combination of solvent properties (both physical and solvation), which are similar to those of DPE (Table 1).

**Figure 6:**
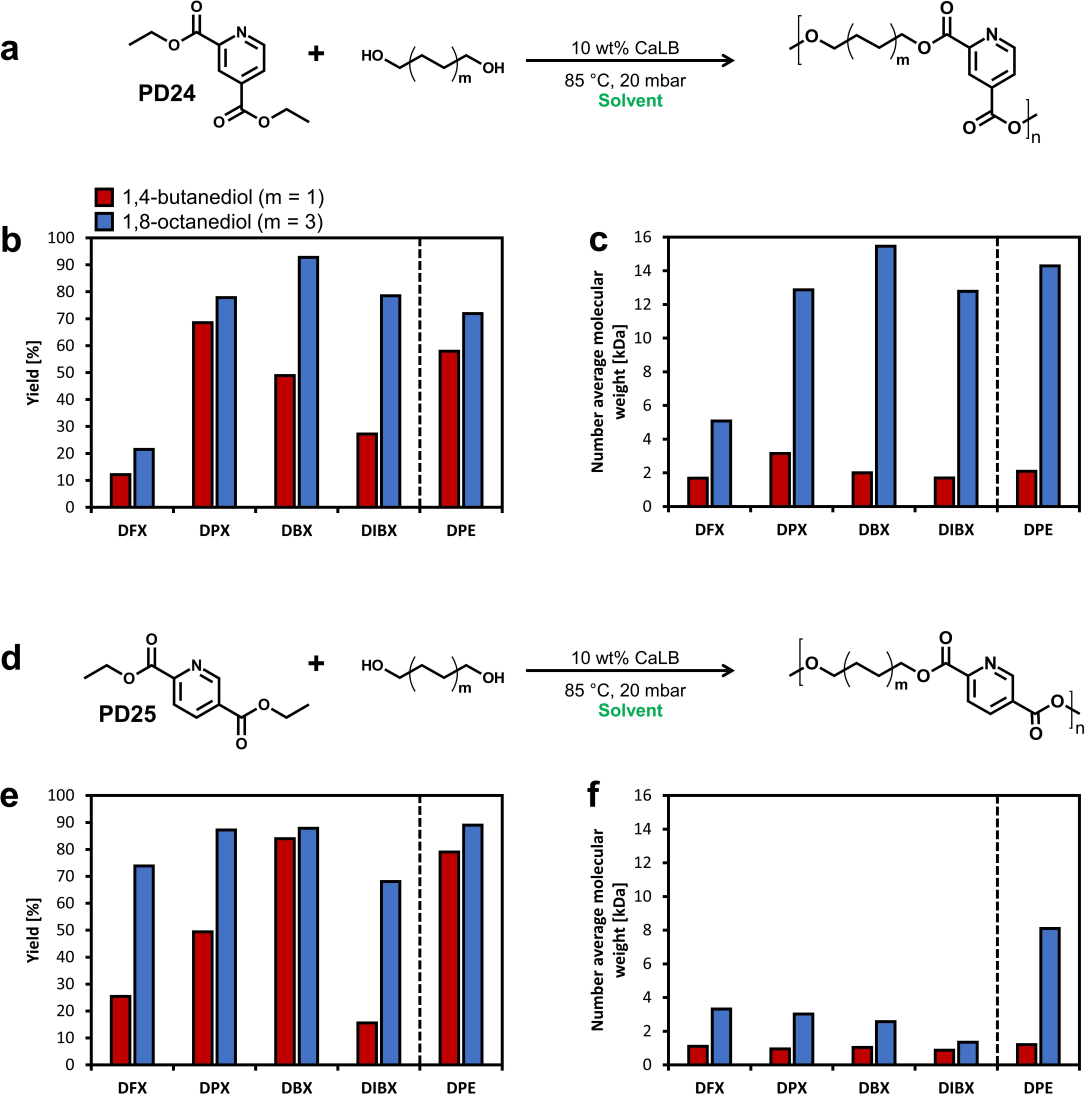
**(a)** Polycondensation reaction scheme of PD24 and diol. **(b)** Yields and **(c)** number average molecular weights of the PD24‐based polyesters produced in xylose acetals and DPE. **(d)** Polycondensation reaction scheme of PD25 and diol. **(e)** Yields and **(f)** number average molecular weights of the PD25‐based polyesters. Values for polyesters produced in DPE were taken from previous works.^[73]^

After the successful synthesis of aliphatic polyesters in xylose acetals, we proceeded to the more challenging polymerisation of aromatic pyridine‐based monomers 2,4‐diethyl pyridinedicarboxylate (PD24) or 2,5‐diethyl pyridinedicarboxylate (PD25) and the same diols (BDO and ODO). PD24 and PD25 can be made from lignin^[72]^ and they have been successfully polymerised with various diols in DPE in the past, providing polyesters with interesting material properties such as rigidity and enhanced stability.[^73^, ^74^, ^75^, ^76^] We produced four polymers, PD24‐BDO, PD24‐ODO, PD25‐BDO, and PD25‐ODO, while using xylose acetals as the reaction media. The yields were over 75 % for ODO‐based polymers and considerably lower and more varied for BDO‐based polymers (Figure 6), which is consistent with previous studies[^71^, ^77^] and the results for aliphatic polyesters obtained in this work. DBX solvent outperformed other xylose acetals as well as the benchmark DPE in terms of yields and molecular weights for most synthesised polyesters. Notably, the polyester produced by the reaction of PD24 with ODO in DBX gave the highest molecular weight (M_n_ of 15.5 kDa and M_w_ of 23.2 kDa). PD25‐based polyesters had considerably lower molecular weights compared to those based on PD24, which is in line with previous studies using these monomers.^[73]^ These results could be explained by limited solubility of both PD25‐based polyesters in all tested xylose acetals even at very low molecular weights, as was evidenced by the precipitation that we observed during the reaction (see SI, Figure S14).

### Structure and Molecular Dynamics of CaLB in Xylose Acetals

**Figure 7:**
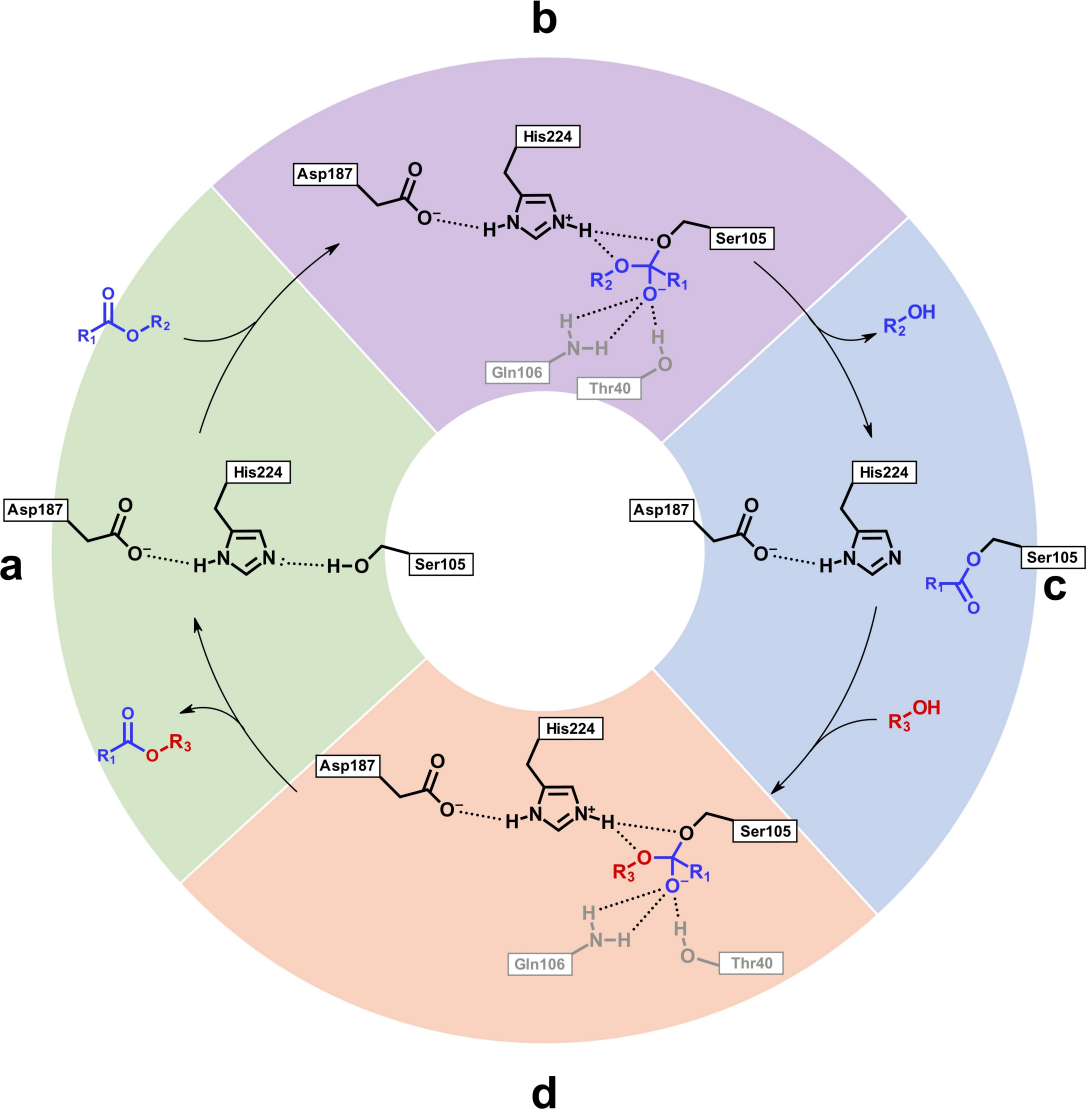
CaLB catalytic cycle of transesterification showing **(a)** the active site in the free enzyme, **(b)** the first reaction intermediate, **(c)** the acylated enzyme complex, and **(d)** the final reaction intermediate. The ester reactant is shown in blue and the diol reactant is shown in red. Residues forming the oxyanion hole are represented in grey, and hydrogen bonds are represented by dotted lines. Adapted from Hevilla et al., 2021.^[79]^

**Figure 8:**
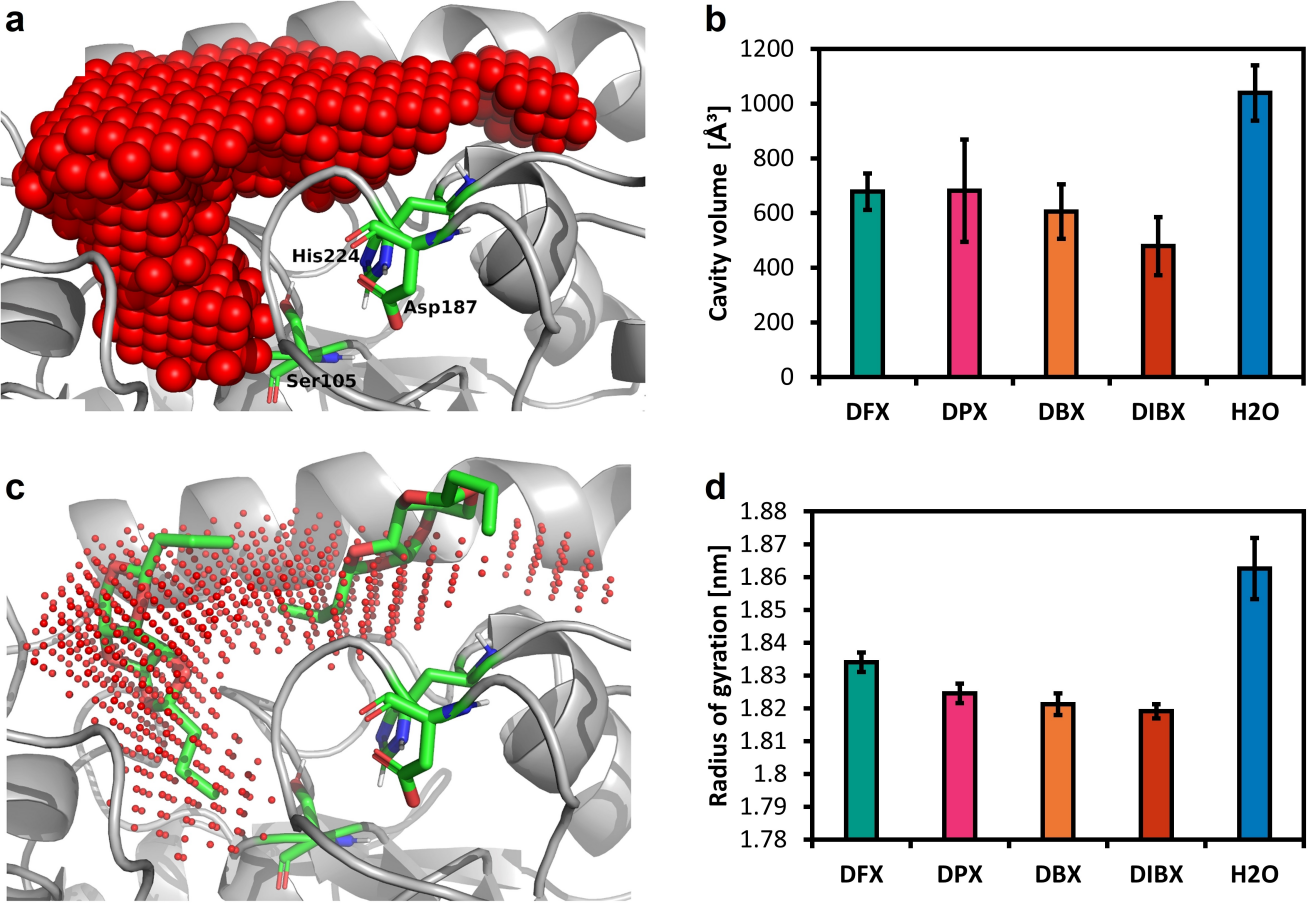
**(a)** Visualised cavity of the CaLB active site in DBX solvent. The cavity is shown as a red point cloud. The active site represented by three amino acids (Ser105, His224, Asp187) is displayed in colour. **(b)** The estimated volume of the CaLB cavity in Å^3^ when the enzyme is exposed to xylose‐based solvent or water. Time dependent graphs for all three replicas can be seen in SI, Figure S17. **(c)** Visualised DBX molecules within the CaLB cavity along with the catalytic triad. Visualised are two molecules that entered the cavity considerably. Three more solvent molecules touched the cavity but are not displayed in this visualisation. **(d)** The radius of gyration shows a compactification of the protein when solvated within xylose‐based solvent compared to water.

To gain insights into the influence of xylose‐based solvents on lipase activity, we performed a series of molecular dynamics (MD) simulations of the CaLB structure exposed to xylose acetals. The active site of CaLB is represented by the catalytic triad (Ser105, His224, Asp187) located in a cavity (Figure 8, a), containing both an acyl binding pocket and an alcohol binding pocket.^[78]^ The active site residues are adjacent to the hydrophobic oxyanion hole which acts to stabilize transition states that arise during the course of the reaction, seen in Figure 7.^[79]^

The first step of the catalytic cycle starts with the abstraction of a proton from the Ser105 residue by His224. This enhances the nucleophilicity of Ser105 and leads to its attack on the carbonyl group of the ester, resulting in the formation of the first reaction intermediate, which is stabilised by hydrogen bonding from residues in the oxyanion hole (Figure 7, b). The second intermediate, or acyl‐enzyme complex, is formed via proton transfer from His224, marking the end of the acetylation step (Figure 7, c). When the diol enters the active site, His224 deprotonates one of its hydroxyl groups, enabling nucleophilic attack of the diol towards the reaction intermediate, and resulting in the formation of the third and final intermediate, seen in Figure 7, d. The newly formed intermediate then collapses, forming an ester bond between the diol and the acyl group, and restoring the enzyme's active site to its original state. The terminal hydroxyl group of this new molecule can now react with another ester molecule, leading to further chain growth of the polyester.^[79]^ Overall, hydrogen bonding within the active site plays crucial role in the overall enzymatic mechanism. In the MD simulation, the hydrogen bond between Asp187 and His224 is present in all tested solvents (see SI, Figure S16). In DPX and DIBX, the hydrogen bond between Ser105 and His224 remains persistent, whereas it appears less frequently in DFX, DBX, and water. Classical simulations fix protonation states and, therefore, we cannot capture the deprotonation of Ser105 due to proton transfer to His224.^[80]^ The increased dynamics in the distance between Ser105 and His224 in both DBX and water suggests that once Ser105 is deprotonated, it becomes flexible enough to perform the nucleophilic attack on the ester, as described above.

To explore how xylose acetals can influence the CaLB cavity, we also analysed the dynamics of the cavity volume in the simulations with these new solvents. The volume of the cavity is defined as the largest contiguous volume close to the active site within the enzyme's convex hull which is not occupied by atoms belonging to the enzyme. The analysis revealed that the cavity in DBX consists of a wide yet shallow region near the surface and another deeper channel within the protein (Figure 8, a). The enzyme cavity can also be split into two separate cavities, with the connection opening and closing flexibly, as we observed for DFX (see SI, Figure S18), possibly hindering binding of the substrates or polymerisation product. The volume of the CaLB cavity is largest in H_2_O and smallest in DIBX, while DBX, DFX and DPX show similar‐sized cavities (Figure 8, b).

The activity of the enzyme can also be significantly affected by the occupation of its cavity by solvent molecules. Specifically, the presence of solvent molecules in the CaLB cavity may hinder its interaction with substrates and interfere with the polymer chain growth. We monitored the occupation of the cavity by xylose‐based solvent molecules in the simulation (Figure 8, c) and found that the average number of heavy atoms (atoms other than hydrogen) per nm^3^ of cavity volume was the highest in the case of DFX (around 50), followed by DPX (40), then DBX (38) and DIBX (38). This result correlates with the molar volume (*V_m_*), β, and π* values decreasing in the same order: DFX>DPX>DBX≥DIBX. Considerably higher number of heavy atoms in the enzyme cavity when exposed to DFX could also possibly contribute to generally lower yields of polyesters produced in DFX from bulky aromatic monomers, for which unhindered access to the active site could be required.

The secondary structure of the enzyme was stable in all tested solvents during the entire simulated time span, according to the analysis with the DSSP (Define Secondary Structure of Proteins) algorithm (see SI, Figure S19). Cluster analysis showed that CaLB maintains a rigid structure with fewer conformations in xylose acetals compared to water (see SI, Table S17), a finding supported by lower radii of gyration in the organic solvents (Figure 8, d). A more rigid enzyme can provide better stability over time as well as higher specificity. Hydrogen bonding analysis of the entire protein confirms the effect of stiffening when exposed to xylose acetals as they boost the number of hydrogen bonds within the protein. In this regard, DBX solvent seems to combine the best of two worlds; while the overall protein stability and compactness is increased, as for all the simulated organic solvents, the hydrogen bonding in the active site allows for sufficient flexibility.

### Recovery and Recycling of Xylose Acetals

**Figure 9:**
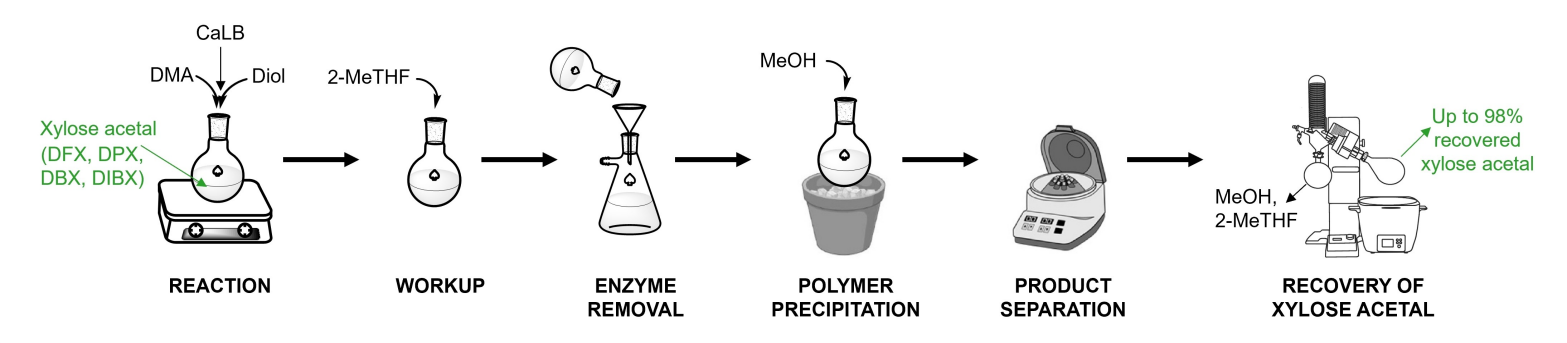
Schematic representation of the enzymatic polycondensation reaction, workup process and solvent recovery.

A fundamental principle of green chemistry is the prevention of waste.^[81]^ Since solvents typically constitute the largest weight component in a chemical reaction, recovering and recycling them becomes even more critical for minimising waste and saving costs. Xylose acetals are high‐boiling solvents with relatively high melting points, which makes traditional distillation methods for their recovery less attractive. We developed an alternative method based on product precipitation to recover xylose acetals after the reaction in the laboratory setting (Figure 9). Upon completion of the polycondensation reaction, we added low‐boiling bio‐based solvent 2‐MeTHF to decrease the overall viscosity of the reaction mixture and facilitate subsequent filtration of the enzyme. After removal of the enzyme, we precipitated the polymers by adding methanol, while keeping xylose acetals in the solution. Centrifugation allowed separation of the polymeric products from the solvents in the supernatant. We then removed the low‐boiling solvents (2‐MeTHF and methanol) from the supernatant by rotary evaporator and successfully recovered up to 98 wt % of xylose acetals with respect to the initial loading. For the implementation of the procedure in industry, separating methanol and 2‐MeTHF would be desirable. Both solvents form an azeotrope with a boiling point of 63 °C, which complicates the separation process. However, methods for separating similar mixtures of methanol and THF using fractional distillation^[82]^ or membranes^[83]^ could potentially be adapted to address this challenge.

The recovered xylose acetals have been applied in two more cycles of the same reaction to explore their reusability. The minor portion of products, ranging from 1–4.5 wt % of the total recovered solvent (as determined from ^1^H‐NMR analysis) was still present in the recovered solvent. This residual content contributed to increased yields of product in the subsequent cycles (see SI, Table S13). Nevertheless, the quantity of polymer detected in the recovered solvent was in the same range after each cycle of the reaction. These results suggest that the xylose acetals serve not only as effective solvents for enzymatic polycondensation but also have the potential for being recycled and reused in subsequent reaction cycles.

## Conclusions

In this work, we introduced a new class of bio‐based aprotic solvents that can be produced from D‐xylose or directly from biomass in high yields using a scalable and sustainable method. The new xylose acetals complement DFX solvent presented in the past work, offering a wider range of physical and solvation properties. Due to the presence of alkyl chains, these solvents are less polar than DFX and their solvation properties are more similar to traditional medium‐polarity solvents that include ethers (THF and 1,3‐dioxolane) and ketones (MIBK, MIAK, and MEK). In addition to being renewably produced, xylose acetals can be advantageous in terms of flammability and safety risks, with higher flash and boiling points as well as remarkable resistance to peroxide formation. However, these new solvent candidates are likely to be acid sensitive similarly to DFX and other carbohydrate‐based solvents such as Cyrene and GVL. Unlike DFX, new candidates DPX, DBX, and DIBX are water‐immiscible liquids at room temperature, which opens new opportunities for their applications and separation. The results presented in this work can serve as a proof of concept for the further development of applications of xylose acetals as a versatile solvent class.

Xylose acetals have proven to be suitable solvents for biocatalytic polycondensation reactions with the lipase CaLB. They facilitated the synthesis of aliphatic and aromatic polyesters with low dispersity in high yields and molecular weights, outperforming the benchmark (but hazardous) solvent DPE in several trials. Computational modelling revealed that these new xylose acetals, despite having only small structural differences, affected the structure and molecular dynamics of CaLB differently, which helped explain varied reaction outcomes. The modelling results aligned with the established model that correlates CaLB activity with three solvent characteristics: polarity, basicity, and molar volume. These properties could be tuned further in the future by introducing a range of other functionalities into the xylose core. Thus, the versatility of the described framework offers opportunities for tailoring solvents to specific applications or enzymes in biocatalysis. Notably, we demonstrated that over 98 wt % of the new high‐boiling xylose‐based solvents can be recovered after the reaction and then successfully reused in subsequent reaction cycles. Therefore, the new xylose acetals offer particular opportunities in sustainable polyester synthesis. Because biocatalysis is often considered a major avenue for improving the sustainability of chemical processes, the successful use of xylose acetals in such applications could further enhance the role of these new solvent candidates in sustainable chemical production.

## Experimental

### Synthesis and Characterisation of Solvents

The synthesis procedure starting from D‐xylose was taken from the previous work for diformylxylose (DFX)^[26]^ and adapted for the synthesis of diethylxylose (DEX), dipropylxylose (DPX), dibutylxylose (DBX), diisobutylxylose (DIBX), and dineopentylxylose (DNPX) as described in detail in SI, Section S3.1. Briefly, D‐xylose and corresponding aldehyde were reacted in 2‐MeTHF with catalytic amount of sulfuric acid at elevated temperature under constant mixing. The quantities of reactants, temperature and time for the synthesis of each molecule are specified in SI, Table S1. After the reaction, the mixture was cooled to room temperature, neutralised, concentrated and purified by crystallisation to obtain white crystals with over 98 % purity. Sensitivity analysis varying temperature, reaction time, and acid concentration was performed to select final reaction conditions. The yields and results of sensitivity analysis are provided in SI, Table S2.

Characterisation of the final structures of xylose acetals was performed using Nuclear Magnetic Resonance (NMR) spectroscopy, X‐ray crystallography, and Gas Chromatography combined with Mass Spectrometry (GC‐MS) and with Flame‐Ionisation Detector (GC‐FID) (see SI for details on analytical methods and Figures S1–S5 for the spectra).

The synthesis procedure starting with corn cobs was adapted from previous work^[28]^ and described in detail in SI, Section S3.2. Briefly, ground and sieved corn cobs were added to 2‐MeTHF in a thick‐walled reagent bottle together with the corresponding aldehyde, followed by the dropwise addition of aqueous solution of HCl (37 wt %). The reaction mixture was heated to 80 °C ensuring sufficient stirring for 30 min. After the reaction, the mixture was cooled to room temperature and filtered to separate cellulose‐rich pulp. A substantial amount of dibutyl ether was added under constant stirring to precipitate the lignin, which was then filtered. The filtrate was concentrated *in vacuo*, and the resulting oil was purified by either crystallisation or by distillation followed by crystallisation to obtain white crystals with over 98 % purity. Sensitivity analysis varying reaction time and acid concentration was performed to select final reaction conditions. The yields and results of sensitivity analysis are provided in SI, Table S3. A compositional analysis of corn cobs is provided in SI, Table S4.

### Measurement of Solvation Properties

Kamlet‐Abboud‐Taft parameters (π*, β) and the wavelength of maximum absorbance in the Nile red dye for each solvent were determined using a previously published procedure (see SI, Sections S3.4–S3.5 and Figure S6).^[26]^ The parameter α was assumed to be 0 due to the aprotic nature of the test compounds. At least three independent samples were prepared to determine the solvatochromic parameters.

Hansen Solubility Parameters (δ_d_, δ_p_, δ_h_) for each solvent were predicted with the HSPiP software 5.3.02 using the Yamamoto‐Molecular Break (Y‐MB) method and the corresponding isomeric SMILES of each compound (see SI, Table S5).

### Measurement of Physical Properties of Solvents

Melting points of synthesised xylose‐based solvents were determined using BUCHI B‐545 melting point apparatus with a 1 °C/min heat ramp in a capillary. The melting point was determined as the temperature at which the solid started melting. The experiment was done in triplicate and the result was averaged (see SI, Table S6).

Boiling points of xylose acetals and DMF (for control) were determined by converting the measured vapor temperature at 8 mbar to boiling point at standard conditions (1 bar) using a pressure‐temperature nomograph (see SI, Section S3.6). The experiment was repeated in triplicate and the result was averaged (see Table S7).

Flash points of xylose‐based solvents were measured in a closed cup by a standard ASTM D6450 test method in duplicate using MINIFLASH‐FLP‐Touch by Grabner Instruments.

Densities of xylose‐based solvents were measured at 50 °C by weighing samples of a given volume in triplicate using an analytical balance (see SI, Table S8).

The solubility of solvents in water was measured at room temperature (25 °C) using HPLC and calibration curve method (see SI, Section S3.7).

To measure the miscibility of DFX and DBX in 16 organic solvents and water, we heated 3 g of DFX and DBX to 50 °C in a glass vial. Then, the same pre‐calculated volume of another organic solvent was added to the vial. The miscibility of two solvents was determined based on visual inspection of the mixture after settling down (see Table S9).

### Peroxide Formation Testing

To test if the synthesised acetal‐stabilised compounds possess any risk of peroxide formation we applied the iodometric titration method. The procedure was taken from the protocol^[84]^ based on European Pharmacopoeia^[85]^ and is described in detail in SI, Section S3.8. The performance of the test procedure was confirmed by analysing a known amount of hydrogen peroxide solution (see Table S10).

### Enzymatic Polycondensation in Xylose Acetals and Solvent Recycling

The synthesis of aliphatic and aromatic polyesters in xylose acetals using CaLB was performed as described in detail in SI, Section S3.9. Briefly, equimolar amounts of diester (DMA, PD24 or PD25) and diol (BDO or ODO) were reacted in xylose acetal (DFX or DPX or DBX or DIBX) with CaLB (10 % by weight of monomers) at 85 °C for 6 h under stirring. Then, the system was placed under a vacuum at 20 mbar for 90 hours. After the reaction, the mixture was filtered while washing with 2‐MeTHF to remove enzyme. Cold methanol was then mixed with the filtrate to precipitate the polyesters. The solution was then centrifuged, and the supernatant was separated and concentrated *in vacuo* to remove the volatile fraction (mostly 2‐MeTHF and methanol), leaving the xylose acetal in the flask. The yields and conversion are provided in SI, Tables S11, S12. The procedure for solvent recovery and recycling was performed as described in detail in SI, Section S3.10. The results are provided in Table S13.

Characterisation of produced polyesters was performed using NMR and GPC. Thermal properties of the synthesised polyesters such as the temperature at which a polymer loses 5 % (*T_d_5*), 10 % (*T_d_10*) or 50 % (*T_d_50*) of its weight at a temperature range from 20–700 °C was determined by Thermogravimetric Analysis. Glass transition temperature (*T_g_*) and melting points (*T_m_*) of polyesters were determined using differential scanning calorimetry (see SI, Section S2 for details on analytical methods and Table S14 for thermal analysis results).

### Molecular Modelling

The crystal structure of the lipase was obtained from PDB Database entry 1TCA.^[86]^ The glycosylation of the protein observed in the PDB‐provided structure was removed and replaced by an oligomannosidic tree. The GROMOS molecular dynamics simulation software^[87]^ together with the 54 A8 force field^[88]^ was used to simulate the protein solvated in five different solvents (H_2_O, DFX, DPX, DBX, DIBX) without ligand for 30 ns each. A Nosé Hoover Chains thermostat was used to keep the temperature at 300 K while the Berendsen barostat kept the pressure at 1 atm. Each simulation was performed in three replicas. The analysis of general protein properties was done using the GROMOS++ molecular dynamics post‐processing software^[89]^ and the in‐house script ProCavTool.

## Author Contributions

A.O.K., C.M.W., A.P., and J.S.L. conceived the project and designed the research. A.O.K. and H.P. conducted the solvent synthesis experiments and characterisations. A.O.K. performed biomass fractionation experiments. C.M.W. conducted the polymer synthesis and solvent recycling experiments, and C.M.W. and A.P. the polymer characterisations. L.K−M. ran the COSMOtherm solvent simulations. J.S. and C.O. ran the GROMOS simulations of solvents and enzyme. A.O.K. and C.M.W. wrote the manuscript. All authors contributed to editing the manuscript. G.M.G., A.P. and J.S.L. acquired the funding. A.P. and J.S.L. supervised the work. A.O.K. and C.M.W. contributed equally to this work and have the right to list their name first in their CV.

## Conflict of Interests

J.S.L. is part owner of Bloom Biorenewables Ltd, a start‐up company that is commercialising the aldehyde functionalisation chemistry of biomass‐derived molecules. J.S.L. and A.O.K are inventors on an International patent (WO/2022/223480) on the use of xylose‐substituted molecules as solvents. The remaining authors declare no competing interests.

1

## Supporting information

As a service to our authors and readers, this journal provides supporting information supplied by the authors. Such materials are peer reviewed and may be re‐organized for online delivery, but are not copy‐edited or typeset. Technical support issues arising from supporting information (other than missing files) should be addressed to the authors.

Supporting Information

## Data Availability

The GROMOS software is available under https://gromos.net/; parameter files for the simulations and the ProCavTool software are available upon request. All other data supporting the findings of this study are available within the article or its supplementary materials.
