# Supplementary material for: Xylose Acetals ‐ a New Class of Sustainable Solvents and Their Application in Enzymatic Polycondensation
Source: ChemSusChem. 2024 Nov 20;18(6):e202401877. doi: 10.1002/cssc.202401877 (PMC11911975; doi:10.1002/cssc.202401877)
Supplement: Supplementary file 1 — Supporting Information [file CSSC-18-e202401877-s001.pdf]

ChemSusChem

Supporting Information

Xylose Acetals - a New Class of Sustainable Solvents and Their Application in Enzymatic Polycondensation

Anastasia O. Komarova, Cicely M. Warne, Hugo Pétremand, Laura König-Mattern, Johannes Stöckelmaier, Chris Oostenbrink, Georg M. Guebitz, Jeremy Luterbacher,* and Alessandro Pellis*

Supplementary Information (SI) for

Xylose Acetals – a New Class of Sustainable Solvents in Enzymatic Polycondensation

Anastasia O. Komarova[1*], Cicely Warne[2,3*], Hugo Pétremand[1], Laura König-Mattern[4], Johannes Stöckelmaier[5], Chris Oostenbrink[5], Georg M. Guebitz[2,3], Jeremy Luterbacher[1**], Alessandro Pellis[3,6**].

[1] Laboratory of Sustainable and Catalytic Processing, Institute of Chemical Sciences and Engineering, École Polytechnique Fédérale de Lausanne (EPFL), Station 6, 1015, Lausanne, Switzerland.
[2] acib GmbH, Konrad-Lorenz-Strasse 20, 3430 Tulln an der Donau, Austria.
[3] Institute of Environmental Biotechnology, Department of Agrobiotechnology, IFA-Tulln, University of Natural Resources and Life Sciences Vienna, Konrad-Lorenz-Strasse 20, 3430 Tulln an der Donau, Austria.
[4] Max Planck Institute for Dynamics of Complex Technical Systems, Process Systems Engineering (PSE), Sandtorstraße 1, D-39106, Magdeburg, Germany.
[5] Institute of Molecular Modeling and Simulation (MMS), University of Natural Resources and Life Sciences Vienna, Muthgasse 18, 1190 Vienna, Austria.
[6] Universitá di Genova, Dipartimento di Chimica e Chimica Industriale, via Dodecaneso 31, 16146, Genova (GE), Italy.

* Joint first authors
** Corresponding authors

E-mail addresses: jeremy.luterbacher@epfl.ch (Jeremy Luterbacher),
alessandro.pellis@boku.ac.at (Alessandro Pellis).

Table of Contents

List of abbreviations 3
S1. Materials and chemicals 4
S2. Analytical methods 4
S3. Experimental procedures and calculations 6
S3.1 Synthesis of xylose acetals from D-xylose 6
S3.2 Production of DFX, DPX, DBX, and DIBX from corn cobs 7
S3.3 Calculation of Biomass Utilisation Efficiency (BUE) 8
S3.4 Measurement of Kamlet–Abboud–Taft (KAT) solvatochromic parameters 9
S3.5 Measurement with Nile Red 10
S3.6 Measurement of boiling points of xylose acetals 10
S3.7 Measurement of water solubility of xylose acetals 11
S3.8 Testing peroxide formation in xylose acetals by iodometry 11
S3.9 Enzymatic polycondensation 12
S3.10 Solvent recovery and recycling 12
S4. Summary of limitations and advantages 13
S5. Supplementary Tables 16
S6. Supplementary Figures 26
References 39

List of abbreviations

BDO	1,4-butanediol
BP	boiling point
CaLB	Lipase B from *Candida antarctica*
$CHCl_3$	chloroform
DBX	dibutylxylose
DIBX	di-isobutylxylose
DFX	diformylxylose
DNPX	dineopentylxylose
DPX	dipropylxylose
DMA	dimethyl adipate
DPE	diphenyl ether
H_2O	water
2-MeTHF	2-methyl tetrahydrofuran
M_n	number average molecular weight
MP	melting point
M_w	weight average molecular weight
NaOH	sodium hydroxide
ODO	1,8-octanediol
PD24	2,4-pyridinedicarboxylic acid
PD25	2,5-pyridinedicarboxylic acid
T_c	crystallisation point
T_d5	temperature at 5% weight loss
T_d10	temperature at 10% weight loss
T_d50	temperature at 50% weight loss
T_g	glass transition temperature
T_m	melting point
V_m	molar volume
α	hydrogen bond donating ability of a molecule
β	hydrogen bond accepting ability of a molecule
δD	energy from dispersion bonds between molecules
δH	energy from hydrogen bonds between molecules
δP	energy from dipolar intermolecular forces between molecules
π	polarizability/dipolarity of a molecule
Đ	dispersity index

S1. Materials and chemicals

1,4-butanediol (ReagentPlus® 99%, Sigma Aldrich), 1,8-octanediol (98%, Sigma Aldrich), diethyl pyridine-2,4-dicarboxylate (Carbosynth), diethyl pyridine-2,5-dicarboxylate (TCI), methanol (for HPLC, gradient grade, >99.9%, Sigma Aldrich), D-xylose (≥99%, Sigma Aldrich), 2-Methyltetrahydrofuran (99% stab. with ca 150-400 ppm BHT, Alfa Aesar), dimethyl adipate (99%, Alfa Aesar), sulfuric acid (95-97%, Supelco), sodium hydroxide (pellets, Reactolab SA), paraformaldehyde (extra pure granules, Carl Roth), dimethyl sulfoxide (>99.5%, Sigma Aldrich), cyclohexane (99%, ABCR), acetaldehyde (≥99.5%, Carl Roth), propionaldehyde (99+%, Acros Organics), butyraldehyde (99+%, Acros Organics), isobutyraldehyde (99+%, Acros Organics), pivaldehyde (>95%, TCI), 4-nitroaniline (99%, Sigma Aldrich), N,N-diethyl-4-nitroaniline (Fluorochem), Nile red (ABCR), Candida antarctica lipase B (CaLB, code: L4777, Sigma Aldrich), hydrochloric acid (37% wt/wt, Merck), dibutyl ether (>99%, ACROS Organics).

S2. Analytical methods

Nuclear Magnetic Resonance (NMR) Spectroscopy

For confirmation of xylose acetal structures, ^{1}H-NMR, ^{13}C-NMR, and 2D-HSQC NMR techniques were applied to the samples dissolved in DMSO-d_6 at 25 °C. Bruker Avance III 400 MHz spectrometer with BBFO-plus probe was used.

For quantification and investigation of polymer structures, ^{1}H-NMR spectroscopy was performed using a JEOL ECZ400R/S3 at a frequency of 400 MHz using $CDCl_3$ as solvent if not otherwise specified.

Gas Chromatography combined with Mass Spectrometry (GC-MS)

The GC-MS was used for confirmation of the xylose acetals structure. Agilent Technologies Gas Chromatography System 7890B with Mass Spectrometer Detector (MSD) with an electron ionization (EI) source 5977A equipped with an Agilent Technologies HP5MS UI Column was used.

Gas Chromatography combined with Flame-Ionization Detector (GC-FID)

The GC-FID was used for the quantification in xylose acetal synthesis. Quantification was performed using standard curves prepared in duplicate and internal standard (1,3-dioxolane) added to each sample (0.075 g per 1 g of sample). The following instrumentation was used: Agilent Technologies Gas Chromatography System 7890B equipped with Flame Ionization Detector and Agilent Technologies HP-5 Column.

High-Performance Liquid Chromatography (HPLC)

The HPLC was used for the quantification of D-xylose in solvent synthesis at the end of the reaction to determine conversion. Quantification was performed using standard curves prepared in duplicate. The following instrumentation was used: Agilent Technologies 1260 Infinity System equipped with refractive index detector, UV-Vis detector, and a BioRad Aminex HPX-87H column at 60 °C with 5mM H_2SO_4 in water at a flow rate of 0.6 mL/min as the mobile phase.

X-ray Crystallography

Single colourless irregular-shaped crystals of DPX, DBX, and DIBX were used. A suitable crystal (0.12 × 0.07 × 0.03 mm^3 dimensions for DPX, 0.08 × 0.08 × 0.05 mm^3 for DBX, 0.13 × 0.05 × 0.03 mm^3 for DIBX) was selected and mounted on an XtaLAB Synergy R, DW system, HyPix-Arc 150 diffractometer. The crystal was kept at a steady T = 140.00(10) K during data collection. The structure was solved with the ShelXT 2018/2[41] solution program using dual methods and by using Olex2 1.5[42] as the graphical interface. The model was refined with ShelXL 2018/3[43] using full-matrix least-squares minimisation on F^2. Crystallographic data are summarized in Supplementary Table 12. The structures were submitted to the Cambridge Crystallographic Data Centre (CCDC) with the following deposition numbers CCDC: 2240423 for DPX S diastereomer, 2240424 for DBX S diastereomer, 2285813 for DBX R diastereomer, and 2240425 for DIBX S diastereomer.

Gel Permeation Chromatography (GPC)

Polyesters were dissolved in $CHCl_3$ to a concentration between 2 and 2.5 mg/mL and filtered through cotton. The analysis was performed at 30 °C on an Agilent Technologies HPLC System (Agilent Technologies 1260 Infinity) connected to a 17,369 6.0 mm ID × 40 mm LHHR-H, 5 µm Guard column and a 18,055 7.8 mm ID × 300 mm L GMHHR-N, 5 µm TSK gel liquid chromatography column (Tosoh Bioscience, Tessenderlo, Belgium) using $CHCl_3$ as an eluent (at a flow rate of 1 mL min^{-1} for 20 min). An Agilent Technologies G1362A refractive index detector was employed for detection. Linear polystyrene calibration standards (250 – 70000 Da) purchased from Sigma-Aldrich were used to calculate the molecular weights of the polymers.

Thermogravimetric Analysis (TGA)

TGA was performed on a Netzsch TG 209 F1 instrument. Samples (1 – 8 mg) were analysed in an Al_2O_3 crucible from 20 to 700 °C, with a heating rate of 10 K/min and a cooling rate of 30 K/min under a N_2 atmosphere.

Differential Scanning Calorimetry (DSC)

Samples between 1.5 – 7 mg were weighed out into concave aluminium pans and measurements were performed on a Netzsch DSC 214 polyma instrument. For aliphatic polymers, a heating rate of 10 K/min was used and the temperature range analysed was -40 to 250 °C. For aromatic polymers, a heating rate of 5 K/min was used and the temperature range analysed was -40 to 200 °C. Two cycles were completed under a N_2 atmosphere for all polymers.

S3. Experimental procedures and calculations

S3.1 Synthesis of xylose acetals from D-xylose

D-xylose (100 g, 0.66 moles, 1 mol. eq.) was added to 2-MeTHF (200 mL) together with 2 mols of the corresponding aldehyde (3 mol eq. to xylose). Then, concentrated sulfuric acid 96 wt% was added dropwise under constant stirring. The reaction vessel was placed in an oil bath heated to a specific temperature for a specific reaction time (Table S1 for temperature and time conditions). At the end of the reaction, the reaction mixture was cooled down to room temperature and 1 ml aliquot was taken for analysis to determine reaction yield. The reaction mixture was neutralised with an aqueous NaOH solution until pH = 7. The organic layer was separated and concentrated at a rotary evaporator set at 45 °C until 25 mbar.

To purify the product, the resulting oil was crystallised in the fridge (at +4 °C) overnight and the resulting crystals were filtered while slightly washing with cold ethanol kept at +4 °C to obtain white crystals (≥ 98% pure by GC-FID). Alternatively, the resulting oil can be distilled at 80 °C, under reduced pressure (0.03-0.05 mbar) to obtain a yellowish/white solid with > 95% purity by GC-FID. The distillate crystallises in the fridge overnight and the crystals can be filtered while slightly washing with cold ethanol kept at +4 °C to obtain white crystals (≥ 98% pure by GC-FID). In both purification procedures, the filtrate was concentrated at a rotary evaporator set at 45 °C and 30 mbar to remove ethanol and repeat crystallisation two more times to isolate all crystal portions. The yields are provided in Table S2. The NMR spectra, GC chromatogram, GC-MS spectra, and single crystal structure of synthesised compounds are provided in Figures 1-5.

DFX: ^{1}H NMR (400 MHz, DMSO) δ 5.97 (d, J = 3.8 Hz, 1H), 4.98 (d, J = 5.8 Hz, 2H), 4.84 (d, J = 6.3 Hz, 1H), 4.63 (d, J = 6.4 Hz, 1H), 4.38 (dd, J = 3.7, 1.0 Hz, 1H), 4.30 (d, J = 2.3 Hz, 1H), 4.07 – 3.98 (m, 1H), 3.93 (tt, J = 2.1, 0.9 Hz, 1H), 3.85 (dd, J = 13.2, 2.1 Hz, 1H); **^{13}C NMR** (101 MHz, DMSO) δ 104.27, 95.82, 90.59, 83.01, 76.88, 74.70, 65.05.
DPX: ^{1}H NMR (400 MHz, DMSO) δ 5.89 (d, J = 4.0 Hz, 1H), 4.88 (t, J = 4.5 Hz, 1H), 4.47 (td, J = 5.3, 1.9 Hz, 1H), 4.38 (d, J = 4.0 Hz, 1H), 4.25 (d, J = 2.1 Hz, 1H), 4.09 – 4.03 (m, 1H), 3.96 – 3.90 (m, 2H), 1.61 (qd, J = 7.5, 4.5 Hz, 2H), 1.47 (td, J = 7.5, 5.3 Hz, 2H), 0.87 (dd, J = 14.7, 7.4 Hz, 6H); **^{13}C NMR** (101 MHz, DMSO) δ 106.52, 104.85, 104.56, 104.36, 99.96, 99.89, 83.77, 83.63, 77.69, 77.60, 74.45, 72.11, 65.36, 65.20, 27.32, 27.27, 27.03, 26.32, 8.11, 7.49, 7.41.
DBX: ^{1}H NMR (400 MHz, DMSO) δ 5.88 (d, J = 4.0 Hz, 1H), 4.91 (t, J = 4.7 Hz, 1H), 4.53 (t, J = 5.2 Hz, 1H), 4.36 (d, J = 4.0 Hz, 1H), 4.24 (d, J = 2.0 Hz, 1H), 4.06 (d, J = 12.8 Hz, 1H), 3.97 – 3.89 (m, 2H), 1.62 – 1.53 (m, 2H), 1.47 – 1.42 (m, 2H), 1.39 – 1.29 (m, 4H), 0.88 (dt, J = 11.5, 7.4 Hz, 7H); **^{13}C NMR** (101 MHz, DMSO) δ 106.15, 105.01, 104.78, 104.51, 99.32, 99.23, 84.14, 84.05, 78.14, 78.05, 74.97, 72.54, 65.84, 65.67, 36.66, 36.60, 36.52, 35.83, 17.21, 17.05, 16.97, 14.27, 14.22.
DIBX: ^{1}H NMR (400 MHz, DMSO) δ 5.88 (d, J = 4.0 Hz, 1H), 4.67 (d, J = 4.6 Hz, 1H), 4.39 (d, J = 4.0 Hz, 1H), 4.27 – 4.23 (m, 2H), 4.09 (d, J = 13.4 Hz, 1H), 3.94 – 3.88 (m, 2H), 1.82 – 1.64 (m, 2H), 0.90 – 0.84 (m, 12H); **^{13}C NMR** (101 MHz, DMSO) δ 109.81, 107.98, 104.95, 102.86, 84.35, 84.12, 78.27, 78.16, 74.84, 72.81, 65.96, 65.81, 32.40, 31.68, 17.39, 17.18, 16.89, 16.82, 16.76, 16.69.

DNPX: ^{1}**H NMR** (400 MHz, DMSO) δ 5.98 (d, J = 3.8 Hz, 1H), 4.99 (d, J = 5.8 Hz, 2H), 4.85 (d, J = 6.4 Hz, 1H), 4.64 (d, J = 6.4 Hz, 1H), 4.39 (dd, J = 3.7, 1.0 Hz, 1H), 4.31 (d, J = 2.3 Hz, 1H), 4.08 – 3.99 (m, 1H), 3.94 (tt, J = 2.1, 0.9 Hz, 1H), 3.86 (dd, J = 13.1, 2.1 Hz, 1H); ^{13}**C NMR** (101 MHz, DMSO) δ 109.65, 104.95, 104.45, 84.22, 78.32, 73.00, 66.01, 34.93, 34.16, 24.88, 24.35.

(GC-MS-EI):

Calculated for DFX with formula $C_7H_{10}O_5$ ($M-H^+$) = 173.15; Found =173.0.
Calculated for DEX with formula $C_9H_{14}O_5$ ($M-H^+$) = 201.20; Found =201.20.
Calculated for DPX with formula $C_{11}H_{18}O_5$ ($M-H^+$) = 229.26; Found =229.20.
Calculated for DBX with formula $C_{13}H_{22}O_5$ ($M-H^+$) = 257.31; Found =257.23.
Calculated for DIBX with formula $C_{13}H_{22}O_5$ ($M-H^+$) = 257.31; Found =257.23.
Calculated for DNPX with formula $C_{15}H_{26}O_5$ ($M-H^+$) = 285.36; Found =285.20.

S3.2 Production of DFX, DPX, DBX, and DIBX from corn cobs

Corn cobs were procured from IP-Suisse in Lausanne, Switzerland. They were sorted to remove residual leaves, stems, and corn. The corn cobs were then ground using a Retsch MS200 cutting mill with a 6 mm screen and sieved with a 0.45 mm and 5 mm mesh. Particles with a size between 0.45 mm and 5 mm were used for solvent synthesis.

Compositional analysis of corn cobs (Table S4) including quantification of sugars (glucan, xylan, arabinan galactan, mannan), Klason lignin, hydration, extractives, and ashes in ground corn cobs was performed using the procedure described in the past work.[1]

For the pretreatment reaction, ground and sieved (0.45 mm – 5 mm) corn cobs (2.5 g) and 8 mL of 2-MeTHF were placed in a 50-mL, thick-walled reagent bottle with an oval PTFE-coated stir bar. Then, the corresponding aldehyde was added to the reactor in the following quantities:

DFX: paraformaldehyde (1.5 g);

DPX: propionaldehyde (1.6 g);

DBX: butyraldehyde (2 g);

DIBX: isobutyraldehyde (2 g).

Then, 0.52 ml of 37 wt% aqueous solution of HCl was added dropwise. The reaction mixture was placed in the oil bath heated to 80 °C ensuring sufficient stirring (400-600 rpm) for 30 min. Then, the reaction mixture was cooled to room temperature, and a 1 ml aliquot was taken and filtered via 0.22 µm PTFE syringe filter to determine reaction yield by GC-FID. The reaction mixture was filtered via a 0.8 µm Nylon filter to separate cellulose-rich pulp while washing with 2-MeTHF (15 ml). The filtrate was transferred to a 250 ml reagent bottle. 80 ml of dibutyl ether was added to the filtrate under constant stirring and the lignin precipitated out. The solution was then filtered via 0.8 µm Nylon filter while washing with di-n-butyl ether to recover lignin and solubilize acetalized sugars. The filtrate was concentrated on a rotary evaporator set at 60-70 °C gradually increasing vacuum from 400 mbar to 25 mbar to remove 2-MeTHF, water, HCl, and di-n-butyl ether sequentially. The resulting brown oil can be crystallised at +4 °C overnight after the addition of the crystal seed. The crystals can be washed with cold ethanol kept at +4 ºC to obtain the pure product (≥ 97% pure by GC-FID). Alternatively, the organic oil can be distilled at 80 °C, under reduced pressure (0.03-0.05 mbar) to obtain a yellowish/white solid with >95% purity by GC-FID. The distillate crystallises in the

fridge overnight and the crystals can be filtered while slightly washing with cold ethanol kept at +4 ºC to obtain white crystals (≥ 98% pure by GC-FID). In both purification procedures, the filtrate was concentrated in a rotary evaporator set at 45 °C and 30 mbar to remove ethanol and repeat crystallisation two more times to isolate all crystal portions. The yields and results of reaction optimization with varying reaction time and acid concentration are provided in Table S3.

S3.3 Calculation of Biomass Utilisation Efficiency (BUE)

BUE shows the percentage of initial biomass ending up in the target product. Stoichiometric BUE (BUE_S) and actual BUE based on the highest reported yield (BUE_H) were calculated for xylose acetals and other selected solvents using the methodology described in detail in the original publication.[2] Briefly, since four hydrogens from the initial xylose molecule (MW 150.1 g/mol) become substituted to form xylose acetal, the BUE_S is 97.3%. BUE_H was calculated by multiplying this BUE_S value by the isolated yield (in mol% on xylan basis) for each xylose acetal.

To calculate the BUE for Cyrene, 2-MeTHF, GVL, and bioethanol we considered the following reactions with the highest reported yields (shown on top of the arrows), assuming 100% efficiency of xylan or glucan separation from biomass and 100% yield of xylose from xylan and glucose from glucan):

2-MeTHF (furfural path)

xylose $\xrightarrow{97\%}$ *furfural + 3 water*

$C_5H_{10}O_5 \rightarrow C_5H_4O_2 + 3\ H_2O$

150 g $\rightarrow$ *96 g + 54 g*

BUE_S = 96 / 150 = 0.64

BUE_H = 0.64 x 0.97 = 0.62

furfural + 4 hydrogen $\xrightarrow{96\%}$ *2-MeTHF*

$C_5H_4O_2 + 4\ H_2 \rightarrow C_5H_{10}O + H_2O$

96 g + 4 x 2 g $\rightarrow$ *86 g + 18 g*

BUE_S = 86 / 96 = 0.90

BUE_H = 0.90 x 0.96 = 0.86

BUE_S (total) = 0.64 x 0.90 x 100% = 58%

BUE_H (total) = 0.62 x 0.86 x 100% = 53%

2-MeTHF (levulinic acid path)

glucose $\xrightarrow{70\%}$ *levulinic acid + formic acid + water*

$C_6H_{12}O_6 \rightarrow C_5H_8O_3 + CH_2O_2 + H_2O$

180 g $\rightarrow$ *116 g + 46 g + 18 g*

BUE_S = 116 / 180 = 0.64

BUE_H = 0.64 x 0.70 = 0.45

levulinic acid + 3 hydrogen $\rightarrow$ *2-MeTHF + 2 water*

$C_5H_8O_3 + 3\ H_2 \rightarrow C_5H_{10}O + 2\ H_2O$

116 g + 6 g $\rightarrow$ *86 g + 36 g*

BUE_S = 86 / 116 = 0.74

BUE_H = 0.74 x 0.92 = 0.68

BUE_S (total) = 0.64 x 0.74 x 100% = 47%

BUE_H (total) = 0.45 x 0.68 x 100% = 31%

GVL (furfural path)

xylose $\xrightarrow{97\%}$ *furfural + 3 water*

$C_5H_{10}O_5 \longrightarrow C_5H_4O_2 + 3\ H_2O$

150 g $\longrightarrow$ *96 g + 54 g*

BUE_S = 96 / 150 = 0.64

BUE_H = 0.64 x 0.97 = 0.62

furfural + 2 hydrogen $\longrightarrow$ *GVL*

$C_5H_4O_2 + 2\ H_2 \longrightarrow C_5H_8O_2$

96 g + 2 x 2 g $\longrightarrow$ *100 g*

BUE_S = 1.0

BUE_H = 1.0 x 0.9 = 0.9

BUE_S (total) = 0.64 x 1.0 x 100% = 64%

BUE_H (total) = 0.62 x 0.9 x 100% = 56%

GVL (levulinic acid path)

glucose $\xrightarrow{70\%}$ *levulinic acid + formic acid + water*

$C_6H_{12}O_6 \longrightarrow C_5H_8O_3 + CH_2O_2 + H_2O$

180 g $\longrightarrow$ *116 g + 46 g + 18 g*

BUE_S = 116 / 180 = 0.64

BUE_H = 0.64 x 0.70 = 0.45

levulinic acid + hydrogen $\xrightarrow{100\%}$ *GVL + water*

$C_5H_8O_3 + H_2 \longrightarrow C_5H_8O_2 + H_2O$

116 g + 2 g $\longrightarrow$ *100 g + 18 g*

BUE_S = 100 / 116 = 0.86

BUE_H = 0.86 x 1.0 = 0.86

BUE_S (total) = 0.64 x 0.86 x 100% = 55%

BUE_H (total) = 0.45 x 0.86 x 100% = 39%

Cyrene

glucose $\longrightarrow$ *LGO + 3 water*

$C_6H_{12}O_6 \longrightarrow C_6H_6O_3 + 3\ H_2O$

180 g $\longrightarrow$ *126 g + 54 g*

BUE_S = 126 / 180 = 0.70

BUE_H = 0.70 x 0. 50 = 0.35

LGO + hydrogen $\longrightarrow$ *Cyrene*

$C_6H_6O_3 + H_2 \longrightarrow C_5H_8O_2$

126 g + 2 g $\longrightarrow$ *128 g*

BUE_S = 1.0

BUE_H = 1.0 x 0.99 = 0.99

BUE_S (total) = 0.70 x 1.0 x 100% = 70%

BUE_H (total) = 0.35 x 0.99 x 100% = 35%

Bio-ethanol (fermentation)

glucose $\xrightarrow{97\%}$ *2 ethanol + 2 carbon dioxide*

$C_6H_{12}O_6 \longrightarrow 2\ C_2H_5OH + 2\ CO_2$

180 g $\longrightarrow$ *92 g + 88 g*

BUE_S = (92 / 180) x 100% = 51%

BUE_H = 0.51 x 0.97 x 100% = 49%

The highest reported yields were taken from the following literature: furfural from xylose,[3] 2-MeTHF from furfural,[4] levulinic acid (LA) from glucose,[5] 2-MeTHF from LA,[6] GVL from furfural,[7] GVL from LA[8], LGO from glucose,[9] Cyrene from LGO,[10] ethanol from glucose.[2]

S3.4 Measurement of Kamlet–Abboud–Taft (KAT) solvatochromic parameters

The KAT parameter α was assigned as 0 due to the aprotic nature of the test compounds. The KAT parameters π* and β were determined based on the shift in the absorption spectrum of two dyes N,N-diethyl-4-nitroaniline (DENA), and 4-nitroaniline (NA). The dyes were dissolved individually in a selected solvent at three concentrations typically ranging from 10^{-3} to 10^{-4} M. The UV-Vis spectra of these samples, as well as reference compounds (DMSO and cyclohexane), were then measured on a UV-visible scanning spectrophotometer UV-3100PC (VWR) at scan step 0.5 nm and a scan range 190–1000 nm. At least three independent samples were used.

From the resulting spectra, the experimental wavenumber at the maximum wavelength of each probe was determined and normalised π*and β parameters were calculated using the following equations:

$$\pi * = \frac{\nu_{\mathrm{DENA(solvent)}} - \nu_{\mathrm{DENA(cylohexane)}}}{\nu_{\mathrm{DENA\ (DMSO)}} - \nu_{\mathrm{DENA(cyclohexane)}}}$$

$$\beta = \frac{(\Delta\nu_{\mathrm{solvent}} - \Delta\nu_{\mathrm{cyclohexane}}) \times 0.76}{\Delta\nu_{\mathrm{DMSO}} - \Delta\nu_{\mathrm{cyclohexane}}}$$

$$\Delta\nu = \nu_{\mathrm{DENA}} - \nu_{\mathrm{NA}}$$

where v is the experimental wavenumber of the wavelength at the maximum absorbance of the dyes in the corresponding solvents.

For xylose acetals, the samples were preheated to 65 ºC. This elevated temperature could bias the usual comparison between solvents because of the thermosolvatochromic behaviour of the dyes. To address this, we measured UV-Vis spectra of DMSO and cyclohexane at 65 ºC as well and then calculated normalised values using the equations above. Since it has been shown that the KAT parameters of oxygenated non-hydrogen bond donor solvents, like xylose acetals, have little temperature dependence,[11,12] we believe the measurement is reasonably accurate at elevated temperatures but this adaptation should still be considered when comparing solvents.

S3.5 Measurement with Nile Red

The Nile Red dye was dissolved in a solvent at a final concentration ranging from 10^{-3} to 10^{-4} M, and UV-Vis spectra were measured to find the wavelength of maximum absorbance. The following equation was used for calculating the transition energy:

$$E_{NR} = 28590 / \lambda \qquad (\mathrm{kcal} / \mathrm{mol}),$$

where λ is the wavelength (in nm) at the maximum absorbance of the Nile Red in the corresponding solvents. The value 28591 is derived from the following energy-wavelength relationship:

$$E = h \cdot c \cdot N_A / \lambda,$$

where h is Planck's constant ($1.584 \cdot 10^{-37}$ kcal·s), N_A is Avogadro's number ($6.022 \cdot 10^{23}$ mol^{-1}) to convert from per particle to per mole, c is the speed of light ($2.998 \cdot 10^{17}$ nm/s).

S3.6 Measurement of boiling points of xylose acetals

3 g of the compound was placed in a two-neck round bottom flask equipped with an oval PTFE-coated stir bar. A thermocouple connected to a temperature controller was inserted into the flask through the rubber cap. The flask was placed in the oil bath equipped with another thermocouple and stirrer and connected to a vacuum at 8 mbar. Upon gradual heating of the mixture, the position of the thermocouple inside the flask was adjusted to match the level of the condensation ring ensuring accurate and reliable temperature readings. The temperature at which we observed gentle reflux of the liquid as measured by the thermocouple inside the flask was registered as vapour

temperature. After that, a pressure-temperature nomograph was used to convert the measured vapour temperature at 8 mbar to boiling point at standard conditions (1 bar). The experiment was repeated in triplicate and the result was averaged (Table S7). The determined boiling point of DMF by this method was 156 ºC, which is 3 ºC higher than the commonly reported value (153 ºC).

S3.7 Measurement of water solubility of xylose acetals
The solubility of solvents in water was measured at room temperature (25 ºC). First, DFX and DPX were found to have limited water solubility, while DBX and DIBX were practically insoluble in water. We prepared several aqueous solutions of DFX and DPX and used HPLC to create calibration curves. Subsequently, we made an oversaturated aqueous solution by adding 0.01 g of DFX or DPX at a time to 1 g of water until the solid did not dissolve anymore. The solutions were filtered using a 0.22 µm PTFE syringe filter and then injected into the HPLC system to determine the maximum concentration of DFX and DPX in water.

S3.8 Testing peroxide formation in xylose acetals by iodometry
The procedure was taken from the protocol[13] based on European Pharmacopoeia.[14] For the experiment, the following solutions were prepared: (1) saturated potassium iodide by adding the first 10 g of KI in 10 ml of deionized water and then gradually adding 0.5 g of KI at a time until the solution no longer dissolves the solid; (2) starch solution of 1 wt % by adding 0.1 g of soluble starch in 10 ml of deionized water and 0.03 ml of formic acid with heating the solution near boiling under stirring; (3) sodium thiosulfate solution of 0.01 M by adding 0.158 g of $Na_2S_2O_3$ to 100-ml volumetric flask and diluting it with deionized water to the final volume of 100 ml; (4) solvent mix of glacial acetic acid : chloroform (3:2 v/v).

3 g of the sample that was stored over 1 year and never melted was taken from the centre of the vial and placed into a 250 ml Erlenmeyer flask closed with a glass stopper. 50 ml of the solvent mixture was added. Then, 1 ml of saturated KI solution was added and allowed to react for 60 ± 2 seconds while vigorously agitating the solution. 100 ml of water was added. The resulting solution was titrated with $Na_2S_2O_3$ solution with constant stirring until the yellow colour (if any) disappeared. Then, 1 ml of starch solution was added as an indicator to increase test sensitivity. The solution was again titrated with $Na_2S_2O_3$ from a purple to a colourless endpoint. The volumes of $Na_2S_2O_3$ solution spent in both titrations are summed up and used to calculate the peroxide value (PV). A blank titration was also carried out under the same conditions without a test substance. The following equations were used to calculate the amount of peroxides present in the sample:

$$PV = \frac{(V_1 - V_0) \times M \times 1000}{m}$$

where V_1 is the volume of consumed sodium thiosulfate solution in the main test (in ml), V_0 is the volume of sodium thiosulfate solution in the blank test (in ml), M is the molar concentration of the sodium thiosulfate solution (in mol/L), and m is the mass of sample (g).

The determined PV is expressed in milliequivalents (meq) of active oxygen per kilogram of the sample. To convert this value to mmol/kg the PV value (in meq/kg)

must be divided by 2 to account for the two equivalents of active oxygen contributed by each mole of peroxide compound (valence of oxygen is 2):

$$PV\left(\frac{mmol}{kg}\right)=\frac{PV\,(\frac{meq}{kg})}{2}$$

The performance of the test procedure was confirmed by analysing a known amount of hydrogen peroxide solution. The detailed results are provided in Table S10.

S3.9 Enzymatic polycondensation

Equimolar amounts (8 x 10-4 mol) of diester (dimethyl adipate (DMA), 2,4-diethyl pyridinedicarboxylate (PD24) or 2,5-diethyl pyridinedicarboxylate (PD25)) and diol (1,4-butanediol (BDO) or 1,8-octanediol (ODO)) were added to a 25-mL round bottom flask with 2 g of xylose acetal, and 10% by weight of monomers of CaLB. The flask was heated to 85 °C and stirred at 400 rpm for 6 hours. After that, the system was placed under a vacuum at 20 mbar for a further 90 hours. Upon completion of the reaction, the reaction mixture was filtered through cotton and the round bottom flask was washed with 1 mL of 2-MeTHF twice. 35 ml of cold methanol was added to the mixture and the solution was vortexed and then centrifuged at 3700 rpm for 10 min at 4 °C. The supernatant was removed, and 20 mL of ice-cold methanol was added again, repeating the washing step two more times. The yields of polymers were determined based on the mass of isolated polymers with respect to theoretical yield. Conversion was calculated based on the amount of unreacted monomer determined by analysis of ^{1}H-NMR spectra (see Tables S11, S12).

S3.10 Solvent recovery and recycling

The solutions remaining after all the washing steps from the reaction between DMA and diols were combined and the volatile fraction (mostly 2-MeTHF and methanol) was removed by a rotary evaporator set at 45 °C. The remaining fraction was identified as xylose acetal and its purity was determined by ^{1}H NMR. The recovered fraction was reused in the second cycle of the same reaction and the procedure was repeated for the third cycle of the reaction. The amount of recovered solvent was calculated based on gravimetric analysis with respect to the initial solvent loading while accounting for polymer and water impurities. The amount of polymer in recovered solvents is calculated from ^{1}H-NMR spectrum (see Table S13 for results).

S4. Summary of limitations and advantages

Below we summarise the limitations and advantages in terms of practical use of xylose acetals, especially in large-scale industrial settings, along with safety and environmental considerations.

Limitations:

- **High melting point.** This factor limits the use of xylose acetals to applications with reaction temperatures above their melting point (25-50 °C). Additionally, solids often require extra steps like melting, grinding, or dissolving, which can add complexity and cost to the operation on a large scale. Liquids, on the other hand, can be easily pumped, mixed, and transported through pipelines. Handling and storage of solids may also present challenges such as dust formation or clogging.

- **High boiling point.** Industrial operation with solvents having boiling points higher than 200°C generally requires more complex and energy-intensive systems, complicating heat integration and necessitating a high-temperature energy source. Additionally, the high temperature can affect the stability of products, sometimes leading to the need for vacuum distillation which in turn increases energy consumption.

- **The presence of two isomers** in the case of DPX, DBX, and DIBX induces variability in the composition of the solvent as the ratio of isomers may change over time and upon heating. This can affect the consistency, reproducibility, and overall performance of chemical reactions. The separation of isomers is not practical because it is expected to be energy-intensive and costly.

- **Moderate stability towards acids.** We have shown in our past work[16] that exposure to acids, especially at high temperatures, can lead to dehydration of DFX into furanic derivatives and eventually to insoluble randomly crosslinked degradation products “humins”. While DFX significantly outperforms another carbohydrate-based solvent Cyrene in terms of stability, we still advise against the use of strong acids when using DFX and other xylose acetals.

Advantages:

- **Low flammability** (flash point >138°C, boiling point >200°C) and **resistance to peroxide formation** offer a competitive advantage related to the overall safety of xylose acetals compared to common medium-polarity ethers and acetals used in industry. Low vapour loss and inhalation hazards can also contribute to reduced insurance costs, enhancing the cost-effectiveness of these solvents.

- **Good thermal stability**. Thermal decomposition is a critical aspect of solvent stability. To evaluate the thermal stability of xylose acetals, we performed Thermogravimetric Analysis (TGA) of pure DFX as a representative of this class of solvents (**Figure S21, a**). TGA showed no mass loss below 110 ºC, with a continuous decline above this temperature, peaking at 222 ºC, likely due to evaporation given DFX's boiling point of 237 ºC. To clarify, Differential Scanning Calorimetry (DSC) was performed, showing no exothermic peak up to 300 ºC, confirming evaporation over decomposition (**Figure S21, d**). The slight increase in the DSC baseline after 300 ºC might indicate insignificant continuous decomposition. We also observed an endothermic melting peak at 63 ºC, indicating complete melting of the sample, while the beginning of the crystal melting (at 48 ºC) was previously measured using BUCHI B-545 melting point apparatus. Additionally, DFX showed no degradation after 48 hours at 100 ºC, as confirmed by GC-FID and GC-MS analysis. These findings demonstrate that DFX can be used up to approximately 200 ºC, also indicating good thermal stability of this solvent class. For most commercial applications, especially in fields like pharmaceuticals, agrochemicals, coatings, this temperature range is sufficient. Some specific industries—like advanced polymer production, electronics, or certain high-temperature extractions—may require solvents that withstand temperatures above 200°C. For these applications, traditional petroleum-based options are typically used.

- **Good stability under hydrogenation conditions.** DFX remained stable under hydrogenation conditions after being subjected to 40 bars of H_2 at 70 °C for 24 hours in a Parr reactor, suggesting that this solvent class could be used in reactions requiring high hydrogen pressure, elevated temperatures, and/or long reaction time, which is a very desirable property for biomass-derived solvents.

- **Low-risk toxicological profile.** In our past work[16] we showed that DFX is non-mutagenic (both directly and indirectly), based on the performed Ames test. Other extensive *in vitro* and *in vivo* tests would be necessary to provide a robust assessment of this solvent class, but full toxicity testing is beyond the scope of this early proof-of-concept work. Nevertheless, the results indicate that these solvents have the potential to be non-toxic.

- **Economically competitive**. When DFX is produced from corn cobs alongside acetal-stabilised lignin and cellulose, the minimum selling price remains at $1.87/kg, assuming the lowest selling prices of $0.6/kg for lignin and $0.1/kg for cellulose at a production scale of 150 ktonne DFX per year.[15] This price is competitive for bio-based solvents, given that market prices for such solvents range from $0.5 to $5/kg (e.g., Cyrene at $3-5/kg, GVL at $1.5-3/kg, 2-MeTHF at $0.5-3/kg).[15]

Since other xylose acetals are synthesised similarly, using the same chemicals, we anticipate their production costs will be in a comparable range, though potentially slightly higher due to differences in aldehyde prices. For instance, paraformaldehyde, a bulk chemical, costs around $0.7/kg in Europe and India and $1.1/kg in the USA (values taken from ChemAnalyst for the third quarter of 2024).[17] In contrast, butyraldehyde is priced at approximately $1.6/kg in Europe (as of second quarter of 2024),[18] with similar prices for propionaldehyde and isobutyraldehyde. Given that raw materials contribute to less than 20% of the overall production cost for the non-neutralised process[15] used in this work to produce xylose acetals, fluctuations in raw material prices have a limited impact on the final selling price, ensuring cost stability and competitiveness.

- **Minimal environmental impact.** The non-neutralised method used in this work to produce xylose acetals from corn cobs has shown low environmental impact and a net negative global warming potential (GWP) at the factory gate for DFX, based on the life-cycle assessment (LCA) in our previous study.[15] For other xylose acetals, although the synthesis method remains the same, we assume that the environmental impact of aldehydes other than paraformaldehyde used for DFX synthesis would result in higher GWP. Indeed, for propionaldehyde, butyraldehyde, and isobutyraldehyde we can expect increased CO_2 emissions due to their higher molecular complexity and the energy-intensive hydroformylation processes used to produce these aldehydes by reacting fossil-derived olefins with syngas (CO, H_2).[19] However, the contribution of fossil-based paraformaldehyde to the overall GWP for DFX production was as little as 5%.[15] Biogenic carbon embedded in corn cobs—an inevitable waste byproduct of maize production—offsets all CO_2 emissions from DFX manufacturing, suggesting that even with other aldehydes, overall emissions would likely remain relatively low and very likely lower than those of fossil-based solvents.

In summary, xylose acetals offer a range of benefits and trade-offs in terms of physical properties, cost, and performance, as well as considerations for disposal and recycling. Implementing these solvents into existing processes would likely require reformulating and adapting workup and solvent recovery procedures on a “case-by-case” basis. However, bio-based solvents like xylose acetals become increasingly desirable and competitive due to global economic trends, particularly in Europe, with its renewable targets, evolving policies, and limited oil reserves. In this regard, our work supports the shift toward more sustainable chemical manufacturing practices.

S5. Supplementary Tables

Table S1. Experimental conditions for the synthesis of xylose acetals from D-xylose and corresponding yields.

	DFX	DEX	DPX	DBX	DIBX	DNPX
Aldehyde	paraformaldehyde	acetaldehyde	propionaldehyde	butyraldehyde	isobutyraldehyde	pivaldehyde
Aldehyde quantity, g	60	88.1	116	144	144	172
$H_2SO_{4\ (96\%)}$, g	17.3	19.7	12.7	13.6	13.6	24.3
$H_2SO_{4\ (96\%)}$, mol	0.17	0.19	0.12	0.13	0.13	0.24
Temperature and time	80 ºC, 2h	80 ºC, 3h	60 ºC, 3h	80 ºC, 3h	80 ºC, 3h	80 ºC, 2h
Xylose conversion, %	99	98	99	98	97	81
Reaction yield, %	89	81	88	91	85	61
S/R ratio in solution	N/A	N/A	4:1	3:1	3:1	N/A
Isolated yield, %	82	73	83	87	80	55
S/R ratio in isolated crystals	N/A	N/A	5.5:1	6:1	6.5:1	N/A

Table S2. Reaction yields of xylose acetals in mol% on xylose basis and their diastereomer S/R ratio in acetalisation reaction at 60°C and 80°C catalyzed by H_2SO_4. The rows highlighted in grey show conditions in the final procedure.

Molecule	Temperature (ºC)	Reaction time (h)	H_2SO_4 (g / g)	Reaction yield (mol%)	S/R ratio in solution
DFX	60	3	0.03	58	N/A
	80	3	0.03	78	N/A
	80	1	0.05	75	N/A
	80	2	0.05	89	N/A
DEX	60	3	0.03	63	N/A
	80	3	0.03	81	N/A
DPX	60	3	0.03	88	4:1
	80	3	0.03	45	1:1
	80	1	0.05	81	1:1
	80	2	0.05	82	1:1
DBX	60	3	0.03	50	4:1
	80	3	0.03	91	3:1
	80	1	0.05	95	2:1
	80	2	0.05	99	2:1
DIBX	60	3	0.03	54	4:1
	80	3	0.03	85	3:1
	80	1	0.05	35	3:1
	80	2	0.05	42	4:1
DNPX	60	3	0.03	23	N/A
	80	3	0.03	44	N/A
	80	1	0.05	26	N/A
	80	2	0.05	61	N/A

Reaction yield was determined from the samples taken right after the reaction completion and analyzed by integrating peaks of the corresponding compounds on GC-FID chromatograms. The diastereomer ratio was based on integrated peak areas in the GC-FID chromatogram and confirmed by integrating corresponding peak areas of the ^{1}H-NMR spectrum.

Table S3. Yields of xylose acetals (after reaction and after workup) in mol% on xylan basis and their diastereomer S/R ratio in the solution after pretreatment of corn cobs at 80 °C in the presence of varied amounts of HCl 37wt% and at varied reaction time. Highlighted rows represent conditions that were selected for the final procedure.

Molecule	HCl (g / g)	Reaction time (h)	Reaction yield on xylan (%)	Isomer ratio	Isolated yield on xylan (%)
DFX	0.02	0.5	16	N/A	N/A
	0.02	1	34	N/A	N/A
	0.02	2	98	N/A	N/A
	0.07	0.5	98	N/A	78
	0.07	1	99	N/A	N/A
DPX	0.02	0.5	97	3:1	70
	0.02	1	99	2:1	N/A
	0.02	2	95	1:1	N/A
	0.07	0.5	53	1:2	N/A
	0.07	1	39	1:2	N/A
DBX	0.02	0.5	99	4:1	73
	0.02	1	99	3:1	N/A
	0.02	2	99	2.5:1	N/A
	0.07	0.5	98	1:1.5	N/A
	0.07	1	50	1:2	N/A
DIBX	0.02	0.5	99	2:1	83
	0.02	1	99	2:1	N/A
	0.02	2	99	2:1	N/A
	0.07	0.5	89	1:1	N/A
	0.07	1	36	1:1.5	N/A

Here, the concentration of pure HCl in the reaction mixture is shown.

Table S4. The composition of corn cobs measured experimentally using a procedure detailed in previous work[1] and used for calculating yields.

Corn cobs content	% (wt/wt) of the raw biomass
Glucan	30.7
Xylan	25.9
Klason lignin	13.5
Acid soluble lignin	N/M
Hydration	6.3
Extractives	5.5
Arabinan	1.8
Galactan	N/D
Mannan	N/D
Ash	0.6
Acetyl	N/M
**Total**	**84.3**

N/M – not measured; N/D – not detected.

Table S5. Hansen Solubility Parameters of solvents ranked by their distance in 3D Hansen space to the data point of **(a)** DPX, **(b)** DBX, **(c)** DIBX.

(a)

Solvent	Hansen Solubility Parameters (MPa½)			Distance
	δD	*δP*	*δH*	
**DPX**	**16.4**	**6.1**	**4.5**	**0.00**
Tributyl phosphate	16.3	6.3	4.3	0.35
Methyl isoamyl ketone	16.0	5.7	4.1	0.98
Methyl propyl ketone	16.0	7.6	4.7	1.71
Cyclopenyl methyl ether	16.7	4.3	4.3	1.91
Butyl propionate	15.7	5.5	5.9	2.07
Propyl pripanoate	15.5	5.6	5.7	2.22
Methyl iso-butyl ketone	15.3	6.1	4.1	2.24
Isophorone	17.0	8.0	5.0	2.30

(b)

Solvent	Hansen Solubility Parameters (MPa½)			Distance
	δD	*δP*	*δH*	
**DBX**	**16.3**	**5.2**	**3.8**	**0.00**
Methyl isoamyl ketone	16.0	5.7	4.1	0.84
Tributyl phosphate	16.3	6.3	4.3	1.21
Cyclopenyl methyl ether	16.7	4.3	4.3	1.30
Methyl oleate	16.2	3.8	4.5	1.58
Diisobutyl ketone	16.0	3.7	4.1	1.64
Methyl iso-butyl ketone	15.3	6.1	4.1	2.21
Butyl propionate	15.7	5.5	5.9	2.44
Propyl pripanoate	15.5	5.6	5.7	2.52

(c)

Solvent	Hansen Solubility Parameters (MPa^{½})			Distance
	δD	*δP*	*δH*	
**DIBX**	**16.2**	**4.9**	**3.2**	**0.00**
Methyl isoamyl ketone	16.0	5.7	4.1	1.27
Diisobutyl ketone	16.0	3.7	4.1	1.55
Cyclopenyl methyl ether	16.7	4.3	4.3	1.60
Methyl oleate	16.2	3.8	4.5	1.70
Tributyl phosphate	16.3	6.3	4.3	1.79
Methyl iso-butyl ketone	15.3	6.1	4.1	2.34
Fatty acid methyl ester	16.4	2.6	4.5	2.67
Butyl propionate	15.7	5.5	5.9	2.94

Table S6. Melting points of diastereomer mixtures of different S/R ratios for DPX, DBX, and DIBX crystals.

Molecule	Diastereomer S/R ratio	Purity (%)	Melting point (°C)
**DPX**	5.5:1	99	23
	7:1	99	33
	9:1	99	32
**DBX**	6:1	99	24
	8:1	99	30
	1:11	99	48
**DIBX**	6.5:1	98	25
	5:1	97	35
	29:1	98	34

The melting point shows the temperature at which the solid begins to melt.
Purity and diastereomer ratios were determined by GC-FID analysis of crystals dissolved in DMSO.

Table S7. Averaged vapour temperatures measured for solvents at 8 mbar and converted to boiling points with a pressure-temperature nomograph.

Molecule	Pressure (mbar)	Vapor temperature (ºC)	Boiling point at 1 bar (ºC)
DFX	8	96	237 ± 3
DPX	8	97	238 ± 5
DBX	8	95	235 ± 4
DIBX	8	95	235 ± 4
DMF	8	32	156 ± 4

Table S8. Density of xylose acetals measured at 50 ºC.

Molecule	Density (g/ml)	Standard deviation σ
DFX	1.35	0.03
DPX	1.17	0.01
DBX	1.15	0.01
DIBX	1.11	0.03

Table S9. Miscibility table of DFX and DBX with other solvents at 50 ºC. Black rectangles show immiscible solvents. White rectangles show miscible solvents.

Solvent	DFX	DBX
Acetone		
Acetonitrile		
Chloroform		
Cyclopentyl methyl ether		
Cyclohexane	■	
Dimethylformamide		
Dimethyl sulfoxide		
1,4-dioxane		
Di-n-butyl ether	■	
Ethanol		
Ethyl acetate		
Hexane	■	
Isopropanol		
2-Methyltetrahydrofuran		
Tetrahydrofuran		
Toluene		
Water		■

Table S10. Peroxide values of xylose acetals and H_2O_2 standard solutions.

Sample	Storage time	Peroxides (mmol/kg)
H_2O_2 (250 mmol/kg)	fresh standard	233
H_2O_2 (0.34 mmol/kg)	fresh standard	0.41
DFX	3 years	N/D
DFX	22 months	N/D
DPX	22 months	N/D
DBX	22 months	N/D
DIBX	22 months	N/D

N/D – not detected. All xylose-based solvent samples are >98% pure.

Table S11. Results of polymerization reactions between DMA and diols performed in the solvents DFX, DPX, DBX and DIBX using CaLB as the biocatalyst, 2-MeTHF as a work-up solvent, and methanol as an anti-solvent.

Diol	Reaction solvent	Polymer yield (%)[a]	Conversion (%)[b]	Remaining solvent (%)[b]	Mn (Da)[c]	Mw (Da)[c]	Đ[c]	PD[c]
BDO	DFX	58	99	<1	12480	15890	1.27	62.3
	DPX	60	99	<1	11266	13588	1.21	56.3
	DBX	77	99	<1	14860	19188	1.29	50.5
	DIBX	70	100	<1	10740	14453	1.35	53.6
ODO	DFX	95	99	3	13929	18693	1.34	54.3
	DPX	93	99	<1	11488	16632	1.45	44.8
	DBX	93	99	2	20133	25456	1.26	78.5
	DIBX	89	99	<1	11342	16081	1.42	44.2

[a] calculated through gravimetric analysis;
[b] calculated through ^{1}H-NMR analysis;
[c] calculated through GPC analysis.

Table S12. Results of polymerization reactions between aromatic PD24 or PD25 and diols performed in the solvents DFX, DPX, DBX and DIBX using CaLB as the biocatalyst, 2-MeTHF as a work-up solvent, and MeOH as an antisolvent.

	**PD24 REACTIONS**							
Diol	Reaction solvent	Yield (%)[a]	Conversion (%)[b]	Remaining solvent (%)[b]	Mn (Da)[c]	Mw (Da)[c]	Đ	PD[c]
BDO	DFX	12	100	<1	1692	1959	1.16	7.6
	DPX	69	98	<1	3161	4258	1.35	14.3
	DBX	49	N/M	<1	2006	2626	1.31	9.1
	DIBX	27	97	<1	1706	2139	1.25	7.7
ODO	DFX	22	97	<1	5082	6938	1.37	18.3
	DPX	78	99	<1	12876	18482	1.44	46.4
	DBX	93	N/M	<1	15463	23237	1.5	55.8
	DIBX	79	100	<1	12793	19144	1.5	46.1
	**PD25 REACTIONS**							
BDO	DFX	25	96	<1	1102	1246	1.13	5.0
	DPX	49	96	2	956	1085	1.14	4.3
	DBX	84	N/M	<1	1042	1214	1.17	4.7
	DIBX	15	100	<1	870	930	1.07	3.9
ODO	DFX	74	95	<1	3319	4735	1.43	12.0
	DPX	87	96	<1	3020	4345	1.44	10.9
	DBX	88	N/M	<1	2571	3618	1.41	9.3
	DIBX	67	95	2	1350	1641	1.22	4.9

N/M – not measured;
[a] calculated through gravimetric analysis;
[b] calculated through ^{1}H-NMR analysis;
[c] calculated through GPC analysis.

Table S13. Results of recovery and recycling of xylose acetals in three cycles of the polymerization reaction between DMA and diols catalysed by CaLB.

Reaction solvent	Diol	Yield (%)			Recovered pure solvent (%)[a]				Polymer in recovered solvent (%)[b]			
		Cycle 1	Cycle 2	Cycle 3	Cycle 1	Cycle 2	Cycle 3	Average (%)	Cycle 1	Cycle 2	Cycle 3	Average (wt%)
**DFX**	BDO	58	76	102	97	91	97	95	3	3	2.4	3
	ODO	95	98	104	82	87	93	87	1	1.2	1.2	1
**DPX**	BDO	21	110	106	73	92	91	85	4.5	4.3	4.5	4
	ODO	65	107	101	46	95	93	78	1.5	1.9	1.9	2
**DBX**	BDO	77	87	111	97	95	94	95	2.4	4.5	3.8	4
	ODO	93	101	109	98	97	97	97	1.2	2	1.5	2
**DIBX**	BDO	70	-	-	91	-	-	91	3.4	-	-	3
	ODO	89	-	-	88	-	-	88	1.7	-	-	2

[a] calculated through gravimetric analysis, accounting for polymer and water impurities;
[b] calculated through ^{1}H-NMR analysis.

Table S14. Thermal analysis of selected polymers from Tables S11 and S12.

Solvent	Diester	Diol	T_d5 (°C)	T_d10 (°C)	T_d50 (°C)	T_m (°C)	T_g (°C)[a]
DFX	DMA	BDO	338	357	392	47	-
		ODO	269	366	406	63	-
	PD24	BDO	-	-	-	-	8.2
		ODO	-	-	-	-	- 6.7
	PD25	BDO	278	300	344	142	-
		ODO	294	326	370	115	-
DPX	DMA	BDO	348	371	403	48	-
		ODO	225	356	405	63	-
	PD24	BDO	271	303	339	-	20.0
		ODO	213	273	341	-	0.5
	PD25	BDO	242	284	343	135	-
		ODO	265	315	369	100	-
DBX	DMA	BDO	353	371	402	50	-
		ODO	25	226	399	65	-
	PD24	BDO	289	308	340	-	15.3
		ODO	243	318	365	-	- 3.4
	PD25	BDO	231	271	341	135	-
		ODO	250	313	370	116	-
DIBX	DMA	BDO	343	367	401	56	-
		ODO	339	371	404	62	-
	PD24	BDO	265	296	338	-	16.8
		ODO	298	329	365	-	- 0.7
	PD25	ODO	259	303	369	97	-

[a] midpoint of curve taken.

Table S15. Crystallographic data for single crystals of S diastereomer of DPX, DBX, and DIBX and R diastereomer of DBX.

Compound	DPX-S	DBX-S	DBX-R	DIBX-S
**Formula**	$C_{11}H_{18}O_5$	$C_{13}H_{22}O_5$	$C_{13}H_{22}O_5$	$C_{13}H_{22}O_5$
**$D_{calc.}$/ g cm^{-3}**	1.304	1.228	1.248	1.267
***m*/mm^{-1}**	0.86	0.774	0.787	0.798
**Formula Weight**	230.25	258.3	258.3	258.3
**Colour**	colourless	colourless	colourless	colourless
**Shape**	irregular-shaped	prism-shaped	plate-shaped	irregular-shaped
**Size/mm^3**	0.12×0.07×0.03	0.08×0.08×0.05	0.26×0.14×0.04	0.13×0.05×0.03
***T*/K**	140.00(10)	140.00(10)	229.99(10)	140.00(10)
**Crystal System**	monoclinic	monoclinic	monoclinic	orthorhombic
**Flack Parameter**	-0.2(3)	0.04(5)	0.11(9)	0.05(6)
**Space Group**	$P2_1$	$P2_1$	$P2_1$	$P2_12_12_1$
***a*/Å**	8.3020(4)	14.8900(3)	12.3072(3)	9.3592(2)
***b*/Å**	8.1708(3)	8.63099(15)	8.81735(17)	11.2703(3)
***c*/Å**	9.0484(5)	22.8820(5)	13.0474(3)	25.6799(5)
***a*/°**	90	90	90	90
***b*/°**	107.209(5)	108.117(2)	103.855(3)	90
***g*/°**	90	90	90	90
**V/Å^3**	586.31(5)	2794.89(10)	1374.67(6)	2708.76(10)
Z	2	8	4	8
Z'	1	4	2	2
**Wavelength/Å**	1.54184	1.54184	1.54184	1.54184
**Radiation type**	Cu*Ka*	Cu*Ka*	Cu*Ka*	Cu*Ka*
**Q_{min}/°**	5.117	3.123	3.489	3.442
**Q_{max}/°**	74.052	74.952	74.766	74.636
**Measured Refl's.**	6852	44004	18344	36743
**Indep't Refl's**	2213	10797	5372	5383
**Refl's I≥2*s*(I)**	1833	9091	4581	4747
**R_{int}**	0.0408	0.0254	0.0349	0.0353
**Parameters**	147	686	329	333
**Restraints**	1	198	1	0
**Largest Peak/e Å^{-3}**	0.623	0.398	0.256	0.217
**Deepest Hole/e Å^{-3}**	-0.373	-0.404	-0.233	-0.227
**GooF**	1.022	1.025	1.003	1.079
**wR_2 (all data)**	0.1928	0.1196	0.1214	0.1
**wR_2**	0.1849	0.1134	0.1167	0.0962
**R_1 (all data)**	0.0864	0.0622	0.0522	0.0517
**R_1**	0.0772	0.0502	0.0447	0.0428

Table S16. Yields of xylose acetals and two other isolated fractions (cellulose-rich pulp and acetal-stabilised lignin) after processing of corn cobs by the non-neutralised method.

Molecule / fractionation protocol	DFX[a]	DPX	DBX	DIBX
Reaction yield (mol% vs. xylan)	90	97	99	99
Isolated yield (mol% vs. xylan)	78	71	73	83
Isolated yield (wt% vs. raw biomass)[b]	20	16	18	21
Cellulose-rich pulp (wt% vs raw biomass)[c]	39	34	35	35
Acetal-stabilized lignin (wt% vs. raw biomass)[b]	8	11	9	10

[a]The data for DFX are taken from the previous work.[15]
[b]The mass yield is corrected for the mass of water and aldehyde to represent the fraction of the original biomass that ends up in the final product.
[c]Cellulose-rich pulp fraction in addition to cellulose contained ashes (0.6%) and traces of xylan (3.5%).

Table S17. The number of clusters obtained by Gromos++ programs rmsdmat and cluster. The md-trajectory was clustered using a 0.10 nm cutoff.

Solvent	Number of clusters		
	Replica 1	Replica 2	Replica 3
DBX	8	5	7
DFX	10	8	10
DIBX	5	5	5
DPX	8	8	13
H_2O	184	120	82

S6. Supplementary Figures

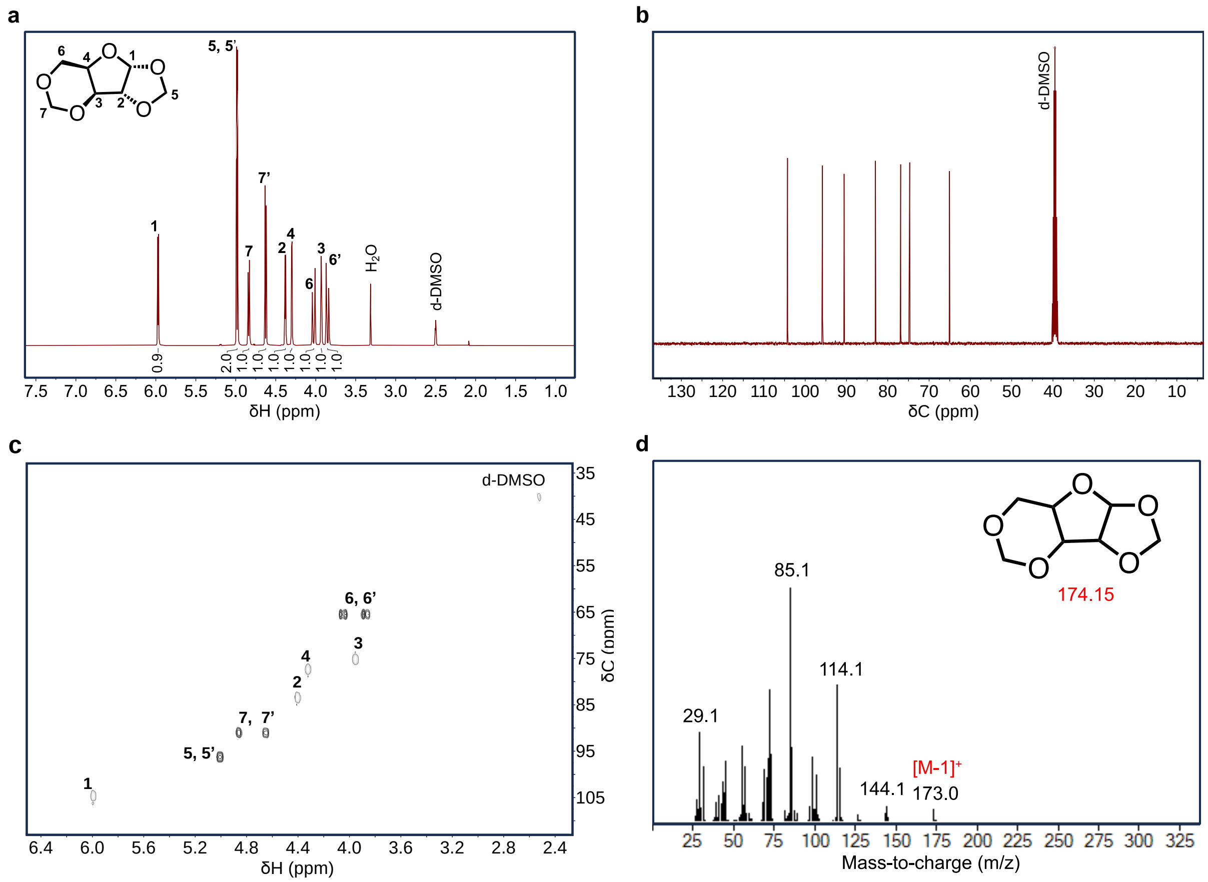

Figure S1. Characterisation of synthesised diformylxylose (DFX). (a) ^{1}H NMR spectrum. **(b)** ^{13}C NMR spectrum. **(c)** 2D HSQC NMR spectrum. **(d)** GC-MS spectrum. The NMR spectra were acquired in DMSO-d_6 at 25 °C.

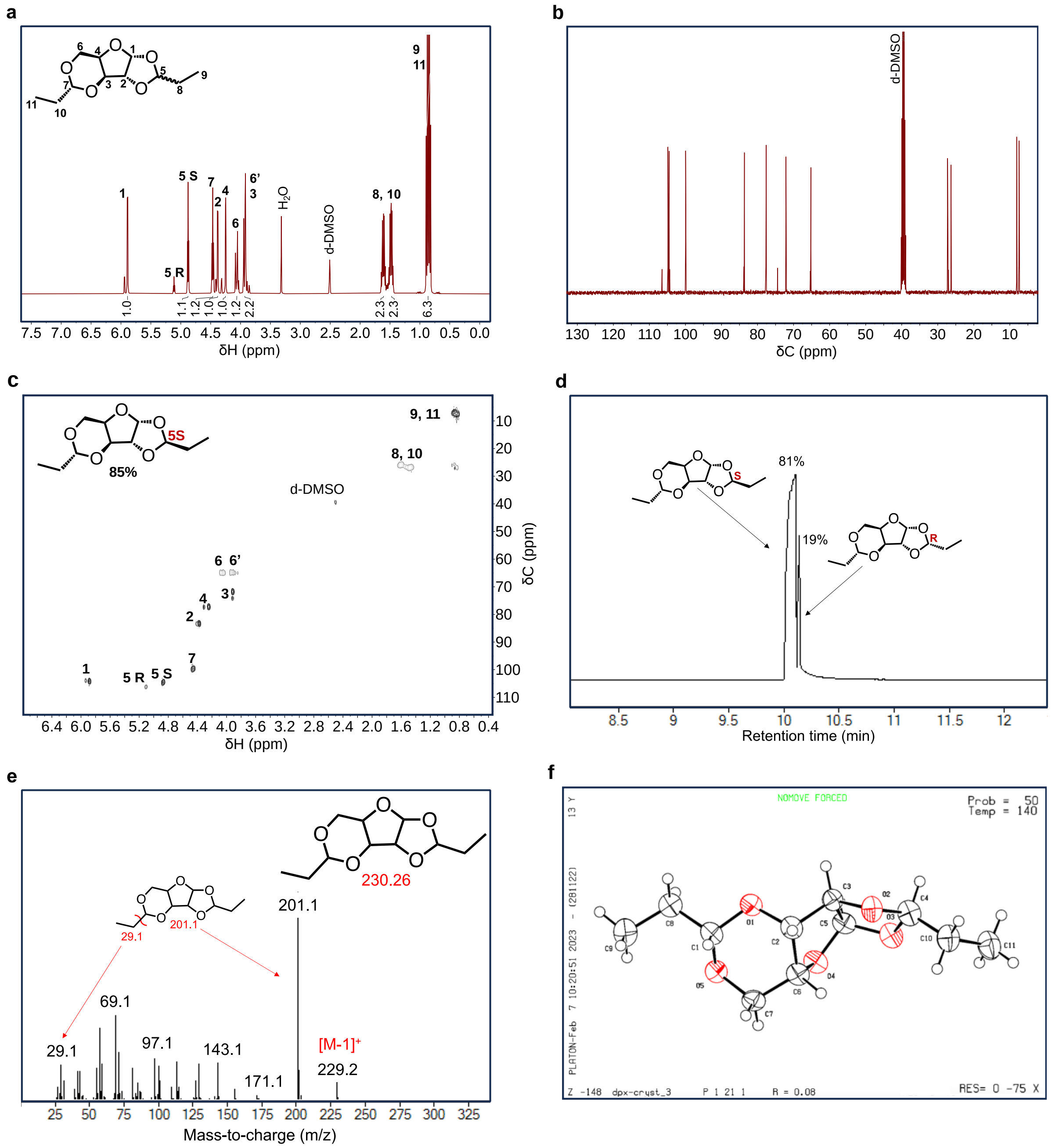

Figure S2. Characterisation of synthesised dipropylxylose (DPX). (a) ^{1}H NMR spectrum, **(b)** ^{13}C NMR spectrum, and **(c)** 2D HSQC NMR spectrum of a sample with diastereomer ratio S/R 5.5:1. (**d)** GC chromatogram with both diastereomers (retention time 10.1 min for S diastereomer). **(e)** GC-MS spectrum (combination of two diastereomers). **(f)** Crystal structure of S diastereomer with probability ellipsoids in the asymmetric unit cell (CCDC 2240423). The NMR spectra were acquired in DMSO-d_6 at 25 °C.

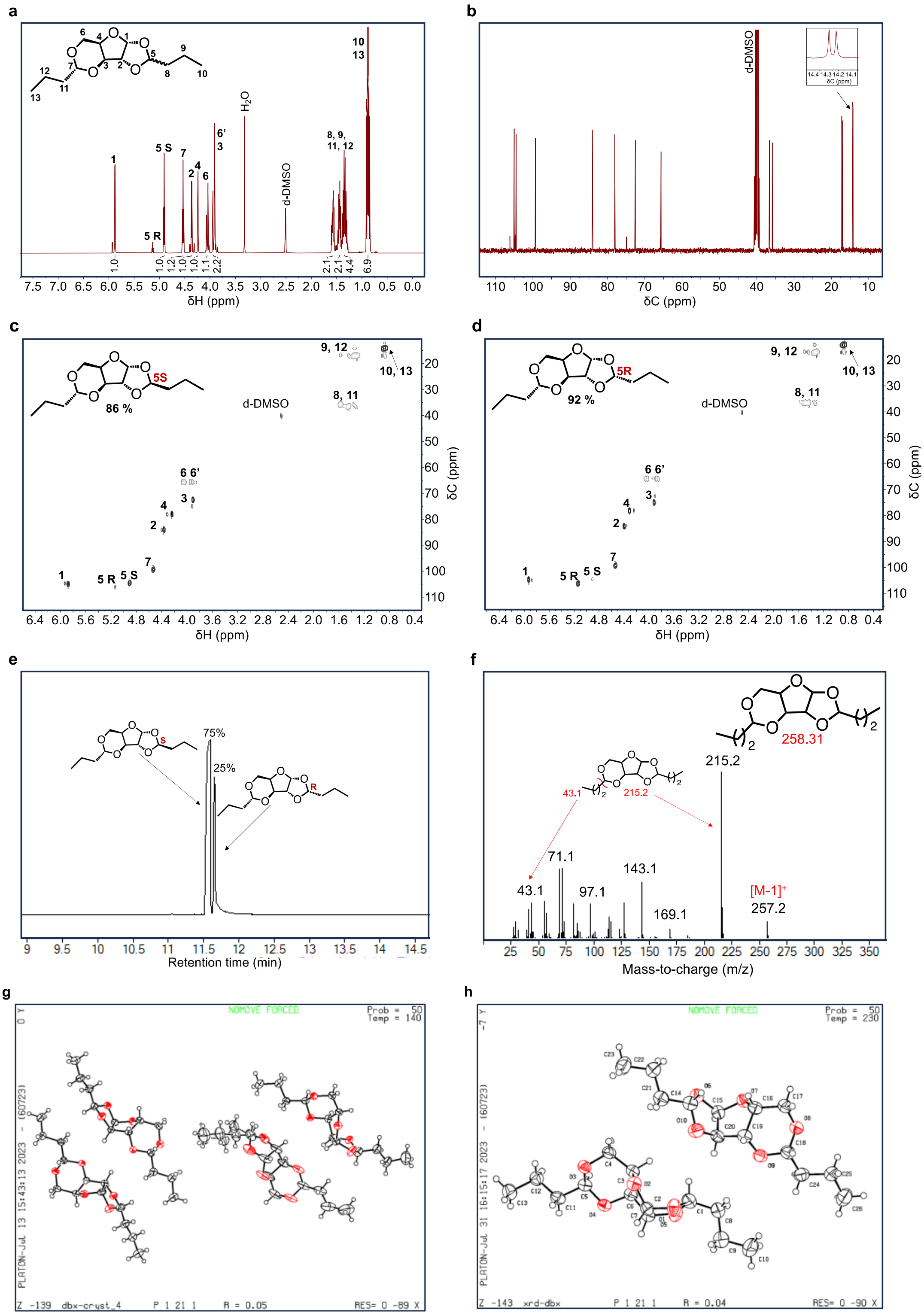

Figure S3. Characterisation of synthesised dibutylxylose (DBX). (a) ^{1}H NMR spectrum, **(b)** ^{13}C NMR spectrum, and **(c)** 2D HSQC NMR spectrum of a sample with diastereomer ratio S/R 6:1. **(d)** 2D HSQC NMR spectrum of a sample with diastereomer ratio S/R 1:11. **(e)** GC chromatogram with both diastereomers (retention time 11.6 min for S diastereomer). **(f)** GC-MS spectrum (combination of two diastereomers). **(g)** Crystal structure of S diastereomer with probability ellipsoids of four conformations in the asymmetric unit cell (CCDC 2240424). **(h)** Crystal structure R diastereomer with probability ellipsoids of four conformations in the asymmetric unit cell (CCDC 2285813). The NMR spectra were acquired in DMSO-d_6 at 25 °C.

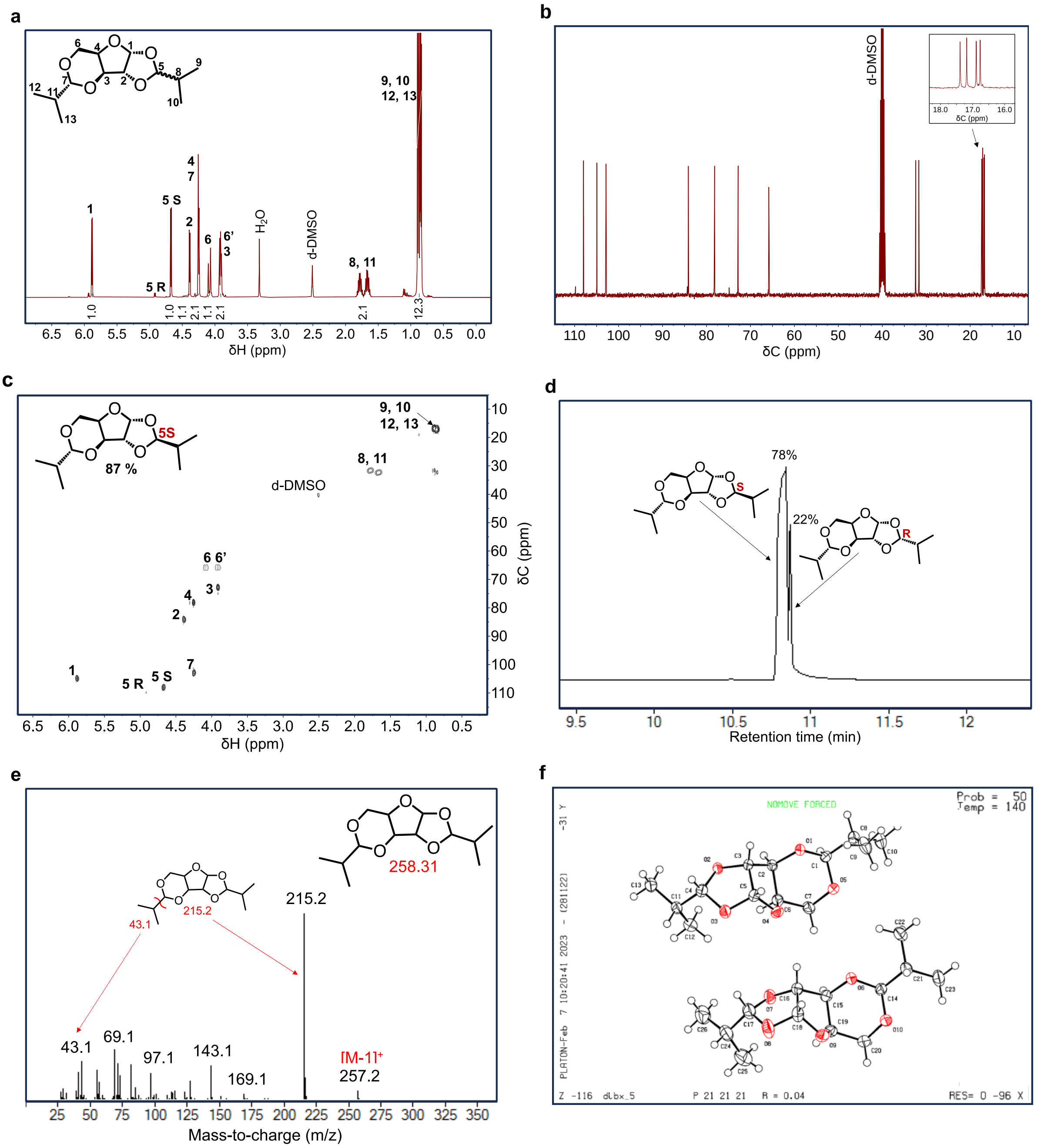

Figure S4. Characterisation of synthesised diisobutylxylose (DIBX). (a) ^{1}H NMR spectrum, **(b)** ^{13}C NMR spectrum, and **(c)** 2D HSQC NMR spectrum of a sample with diastereomer ratio S/R 6.5:1. **(d)** GC chromatogram with both diastereomers (retention time 0.8 min for S diastereomer). **(e)** GC-MS spectrum (combination of two diastereomers). **(f)** Crystal structure of S diastereomer with probability ellipsoids of two conformations in the asymmetric unit cell (CCDC 2240425). The NMR spectra were acquired in DMSO-d_6 at 25 °C.

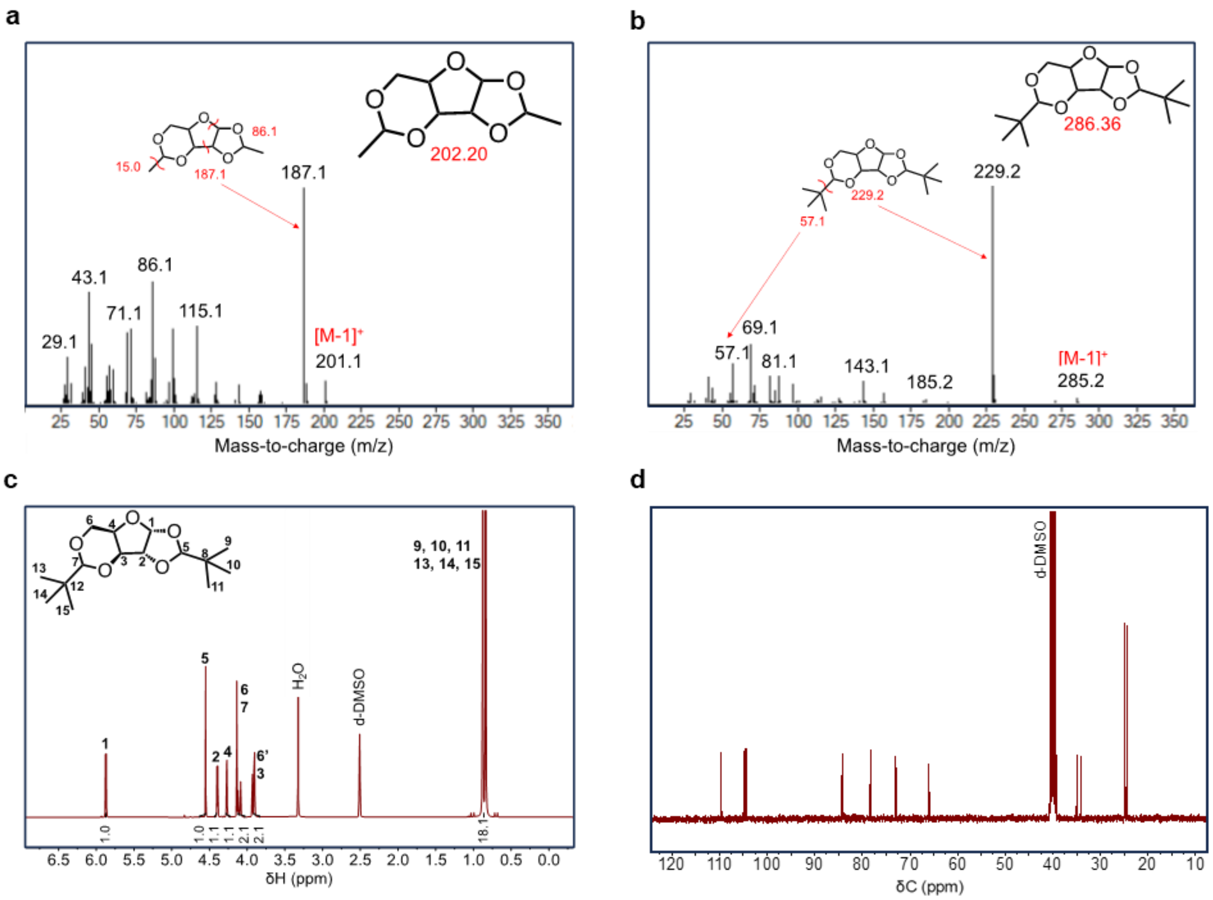

Figure S5. Characterisation of synthesised diethylxylose (DEX) and dineopentylxylose (DNPX). (a) GC-MS spectrum of DEX. **(b)** GC-MS spectrum of DNPX. **(c)** ^{1}H NMR spectrum and **(d)** ^{13}C NMR spectrum of DNPX. The NMR spectra were acquired in DMSO-d_6 at 25 °C.

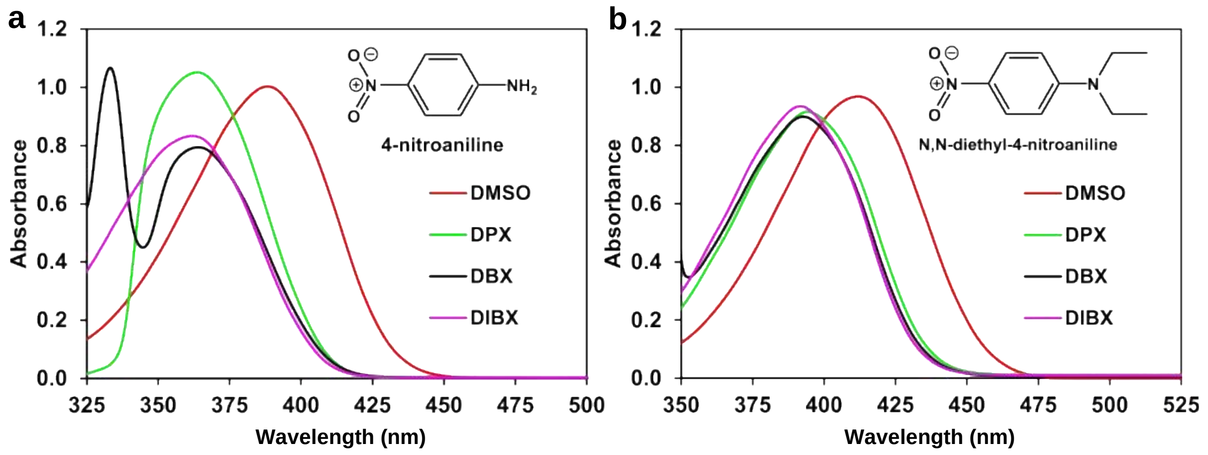

Figure S6. Absorbance spectra of the dyes in xylose acetals. (a) 4-nitroaniline, 4 µM, **(b)** *N,N*-diethyl-4 nitroaniline, 4 µM.

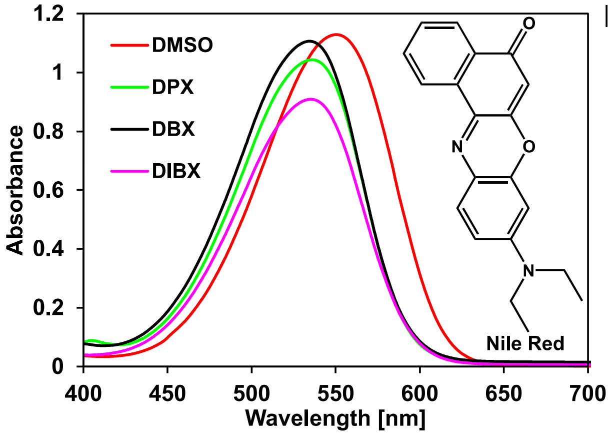

Figure S7. Absorbance spectra of Nile Red (24 µM) in xylose acetals.

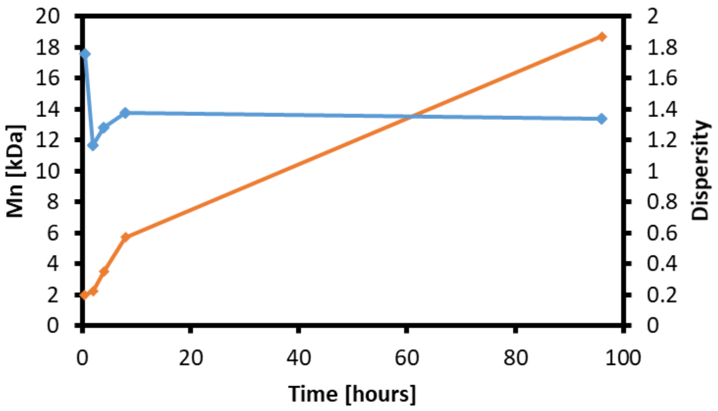

Figure S8. Dispersity (blue line) and Mn (orange line) of poly(octylene adipate) synthesised from DMA and ODO in DFX at different time points as measured by GPC.

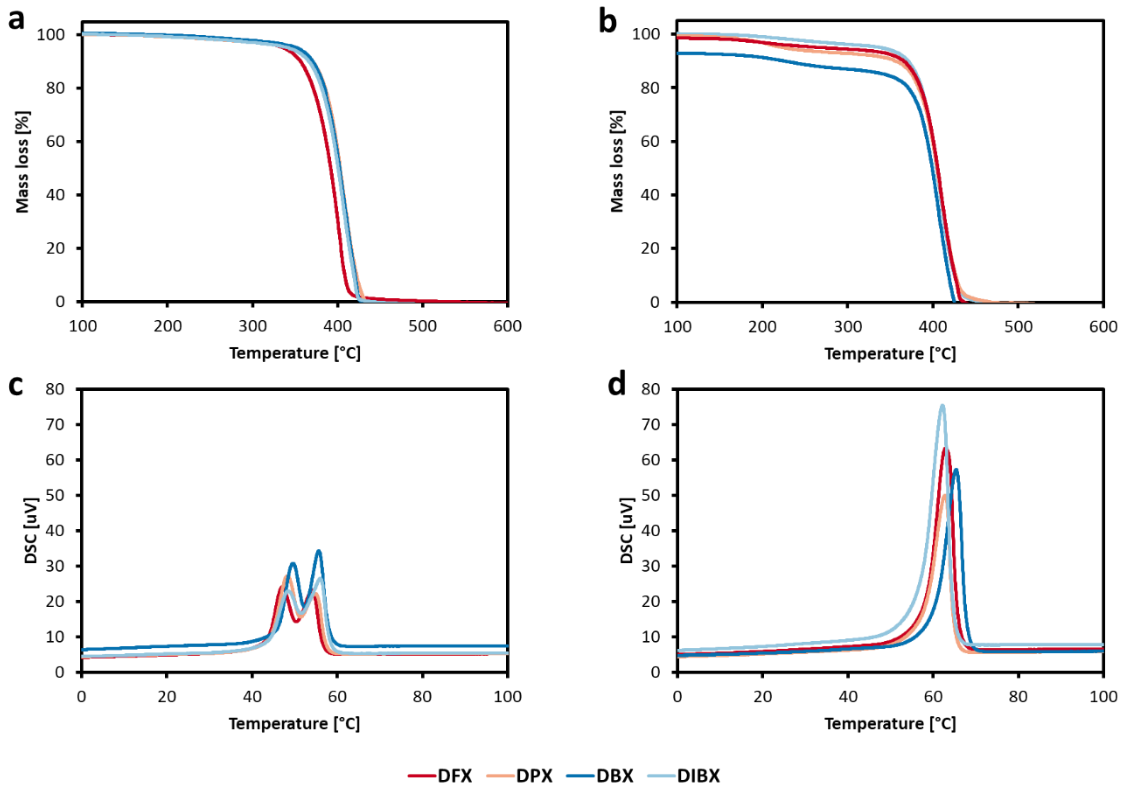

Figure S9. TGA (a, b) and DSC (c, d) curves of aliphatic polymers produced in xylose acetals. (a) TGA of BDO-based polymers, **(b)**TGA of ODO-based polymers, **(c)** DSC of BDO-based polymers, **(d)** DSC of ODO-based polymer. Polyesters synthesised in DFX are shown in red, DPX in light orange, DBX in dark blue and DIBX in light blue.

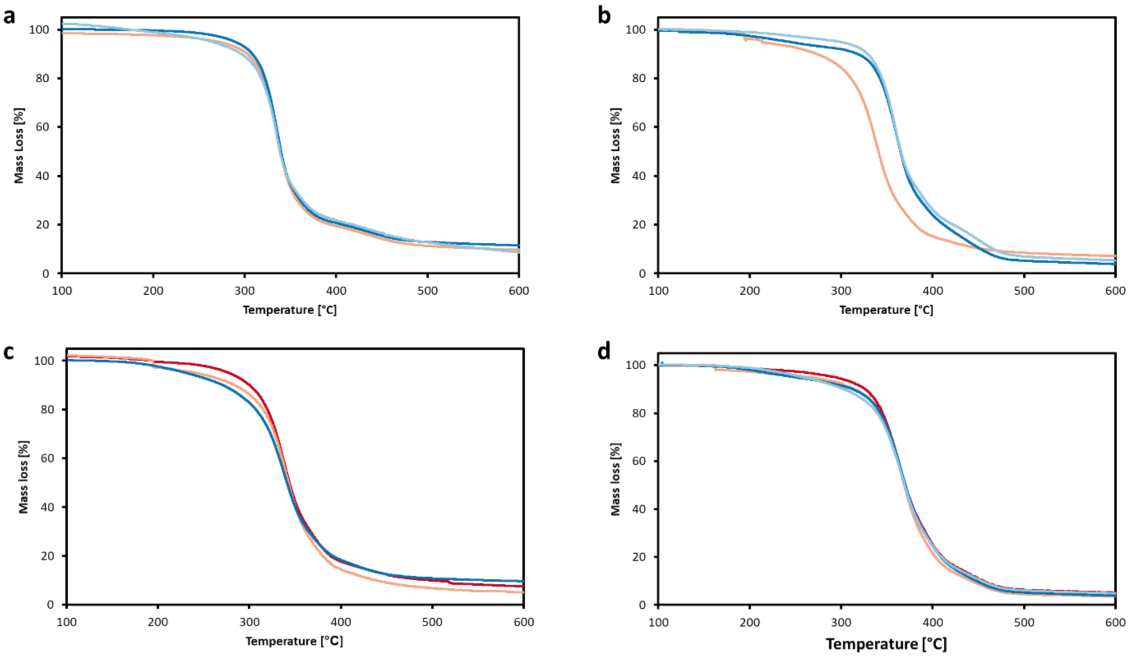

Figure S10. TGA curves of aliphatic polymers produced in xylose acetals. (a) PD24-BDO, **(b)** PD24-ODO, **(c)** PD25-BDO, **(d)** PD25-ODO. Polyesters synthesised in DFX are shown in red, DPX in light orange, DBX in dark blue and DIBX in light blue.

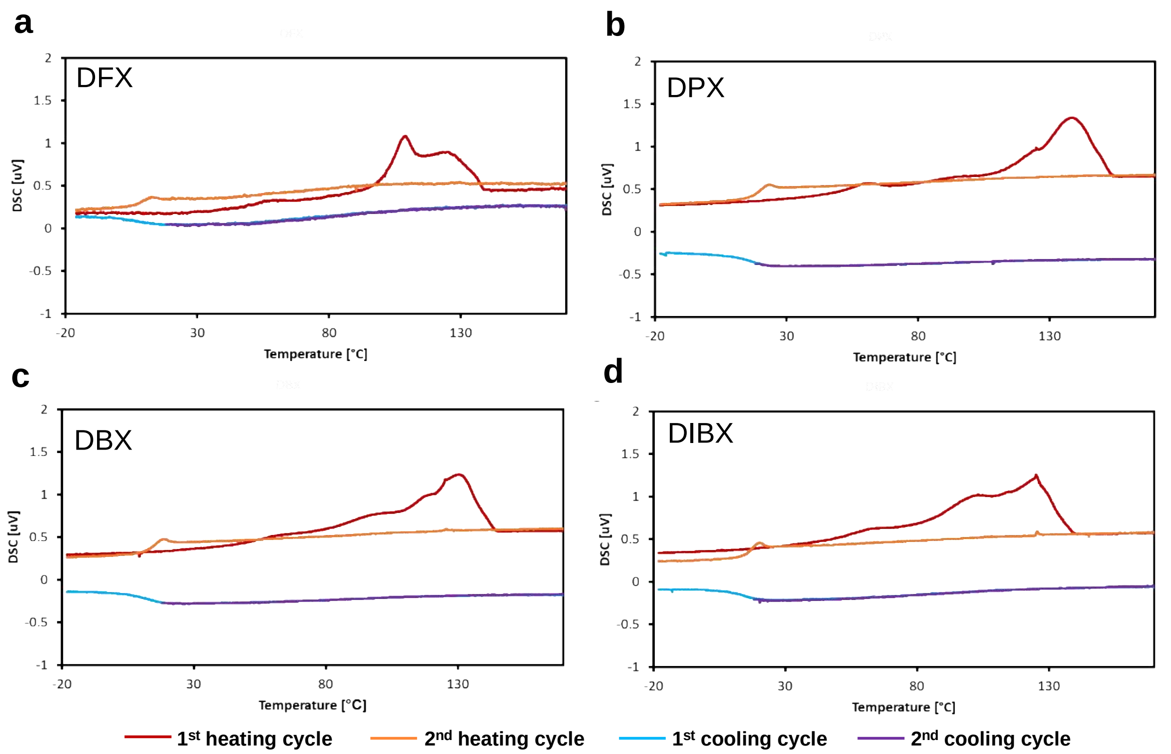

Figure S11. DSC curves of aromatic PD24-BDO polymers synthesised in (a) DFX, **(b)** DPX, **(c)** DBX, and **(d)** DIBX. The first heating cycle is shown in red, the first cooling cycle in blue, the second heating cycle in orange, and the second cooling cycle in purple.

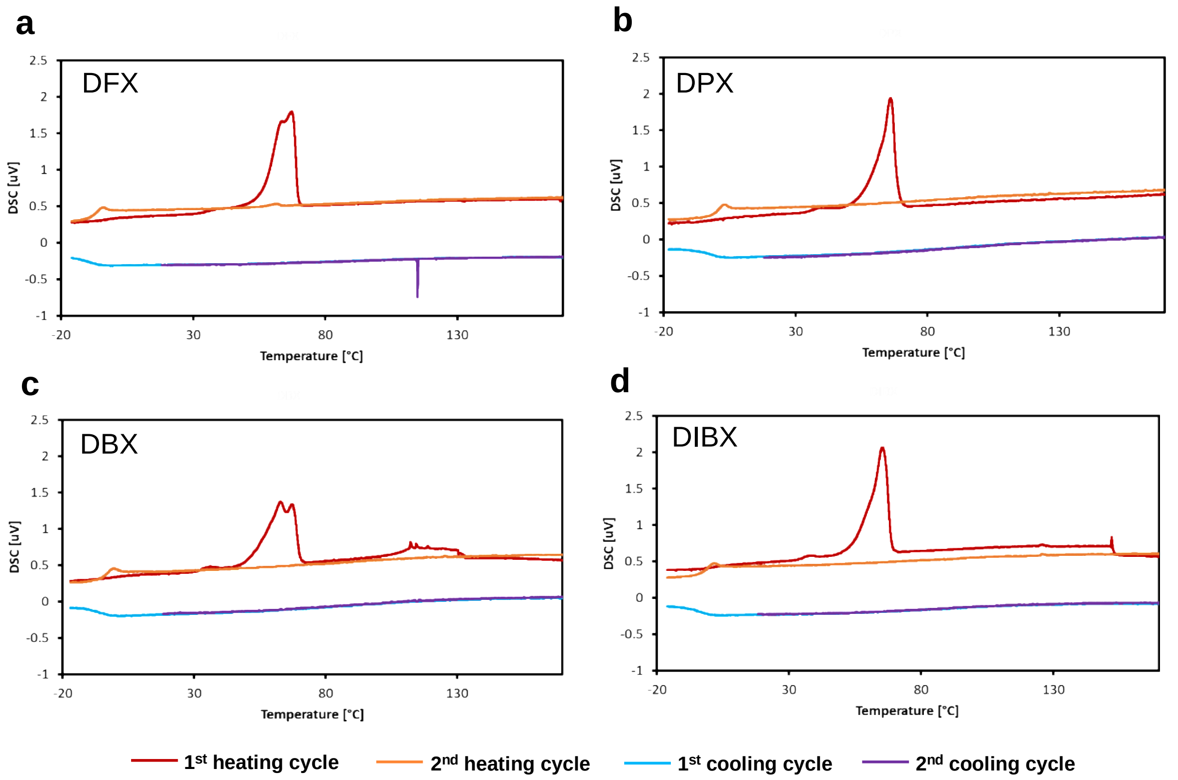

Figure S12. DSC curves of aromatic PD24-ODO polymers synthesised in (a) DFX, **(b)** DPX, **(c)** DBX, and **(d)** DIBX. The first heating cycle is shown in red, the first cooling cycle in blue, the second heating cycle in orange, and the second cooling cycle in purple.

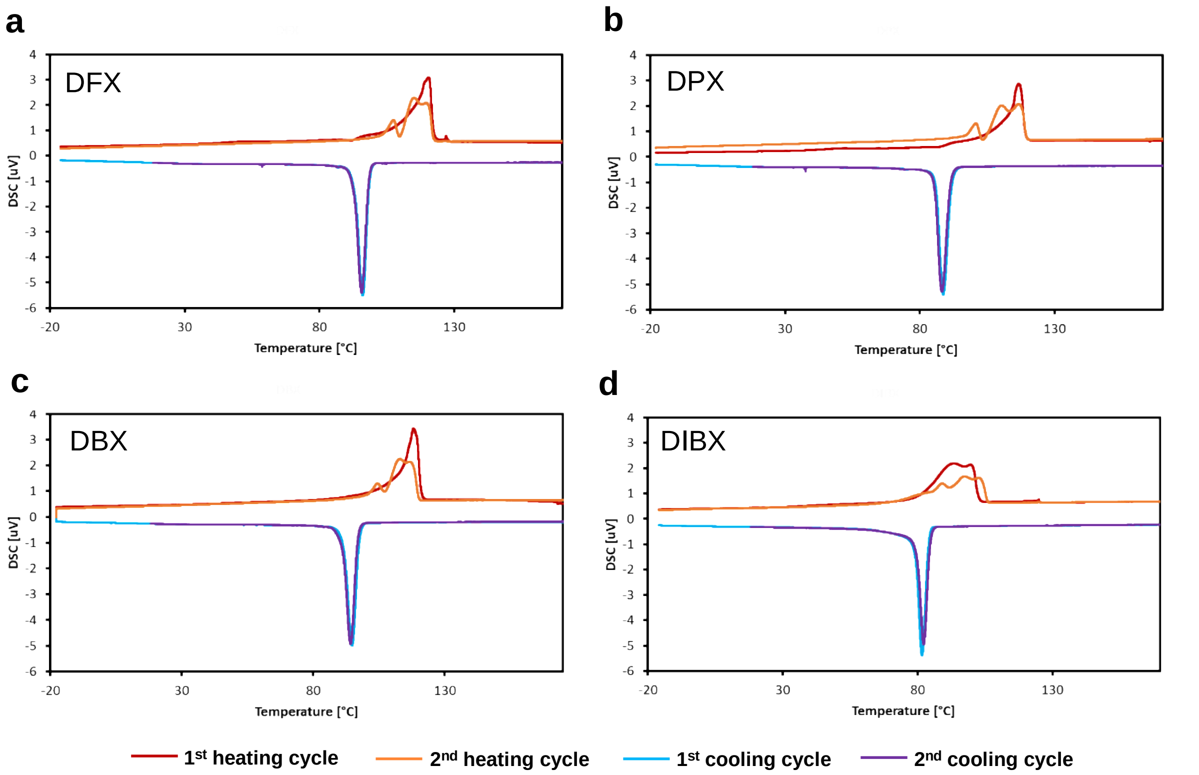

Figure S13. DSC curves of aromatic PD25-ODO polymers synthesised in (a) DFX, **(b)** DPX, **(c)** DBX, and **(d)** DIBX. The first heating cycle is shown in red, the first cooling cycle in blue, the second heating cycle in orange, and the second cooling cycle in purple.

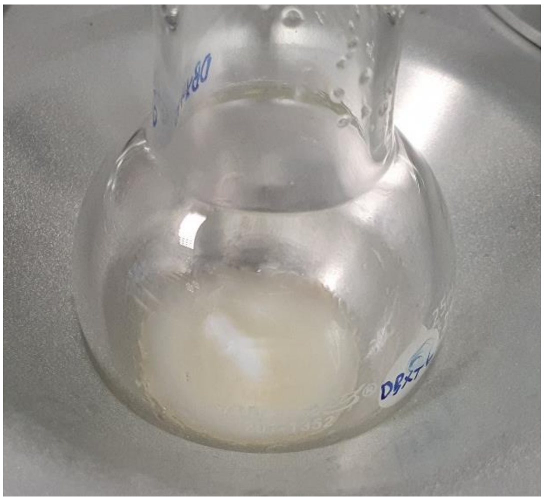

Figure S14. Photo of the reaction mixture with PD25-ODO polymer synthesised in DBX after 72h with visible precipitation and hindered stirring.

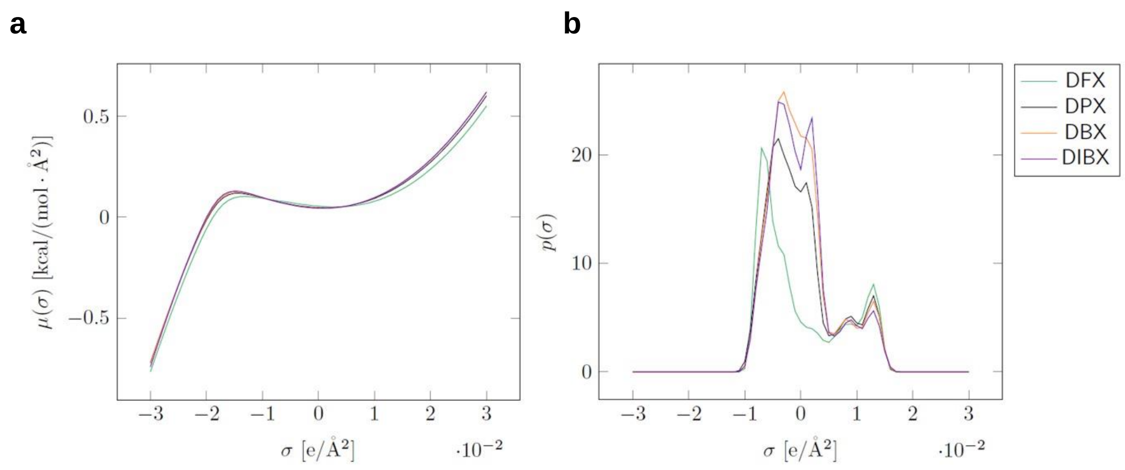

Figure S15. (a) Sigma potential and **(b)** sigma profile of xylose acetals modelled with COSMO-RS.

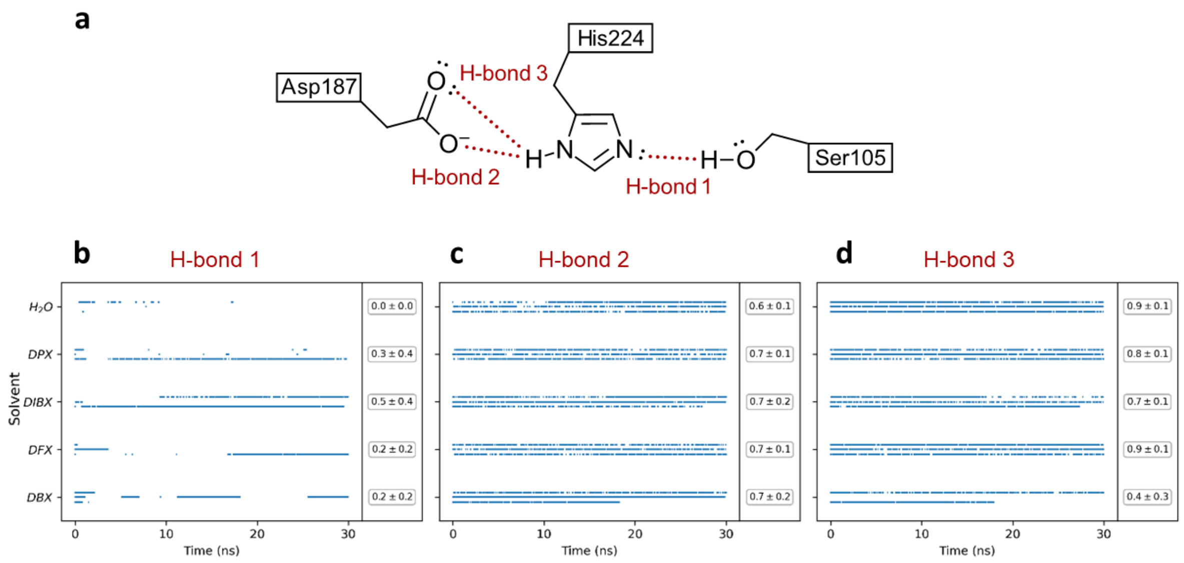

Figure S16. (a) A diagram of the CaLB active site showing three possible hydrogen bonds (1, 2 and 3), and the expectation value of the occurrence of (**b)** H-bond 1, (**c)** H bond 2, and (**d)** H bond 3. A bond of 0.5 can be interpreted as a hydrogen bond existing 50% of time. The hydrogen bonds from histidine to each of the two carbonyl oxygens of aspartic acid can be added up, so that the carbonyl group shows between 1.1 and 1.6 hydrogen bonds to the histidine. For each solvent and hydrogen bond, three lines representing the three replicas are shown. A blue dot represents an existing hydrogen bond at a specific time point**.** To avoid overlapping of the dots and to get an interpretable graphic, dynamic striding was applied. If a simulation showed only a few frames with hydrogen bonds, only little striding was applied to not omit those dots. If nearly all frames showed an existing hydrogen bond, strong striding was applied to avoid overlapping of the dots such that fluctuations of the hydrogen bonds still could be visualized.

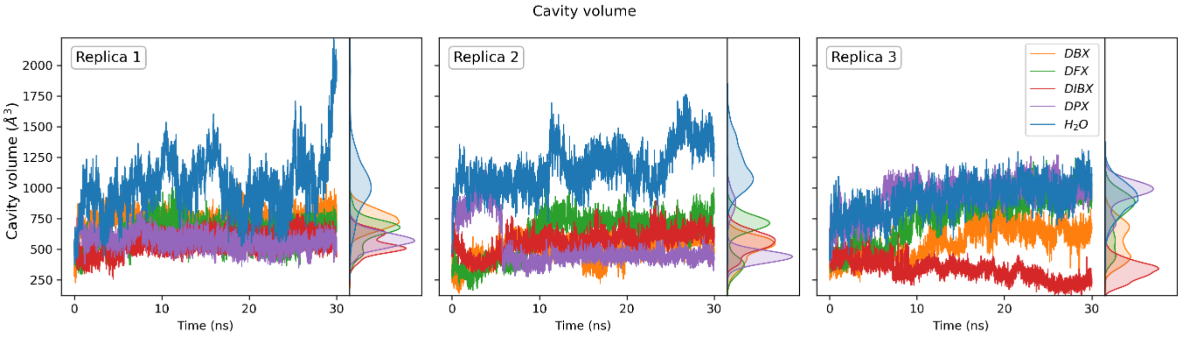

Figure S17. The volume fluctuations during the simulated 30 ns are especially high if the solvent is water.

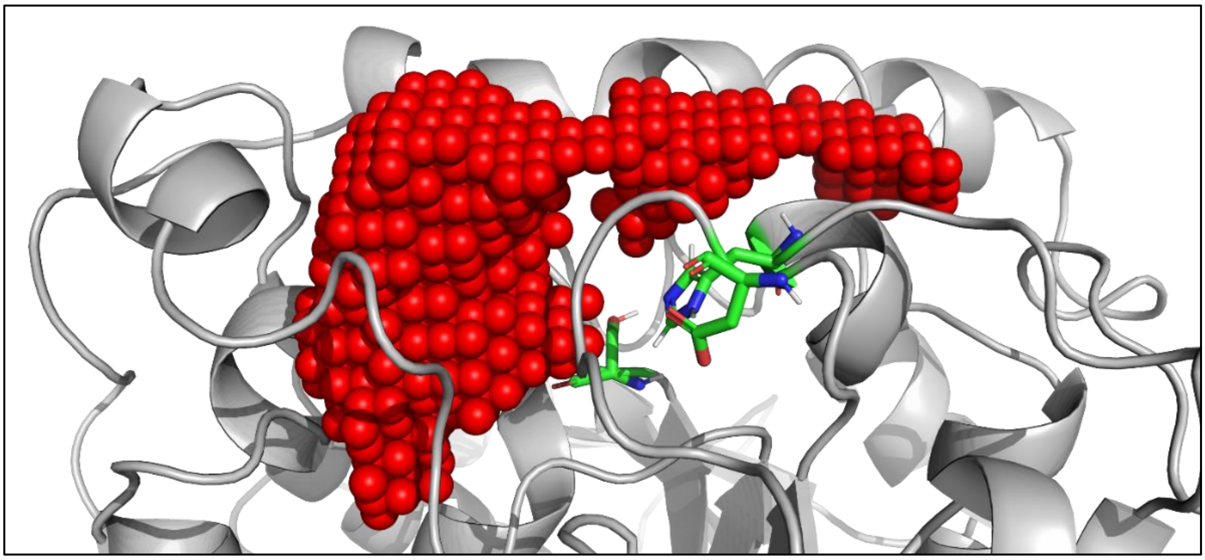

Figure S18. The visualisation of the cavity nearly split in DFX solvent. The shallow part of the cavity, visualised as red point cloud, can be split off from the deep and narrow part. This process is reversible and can fluctuate on a sub-ns timescale.

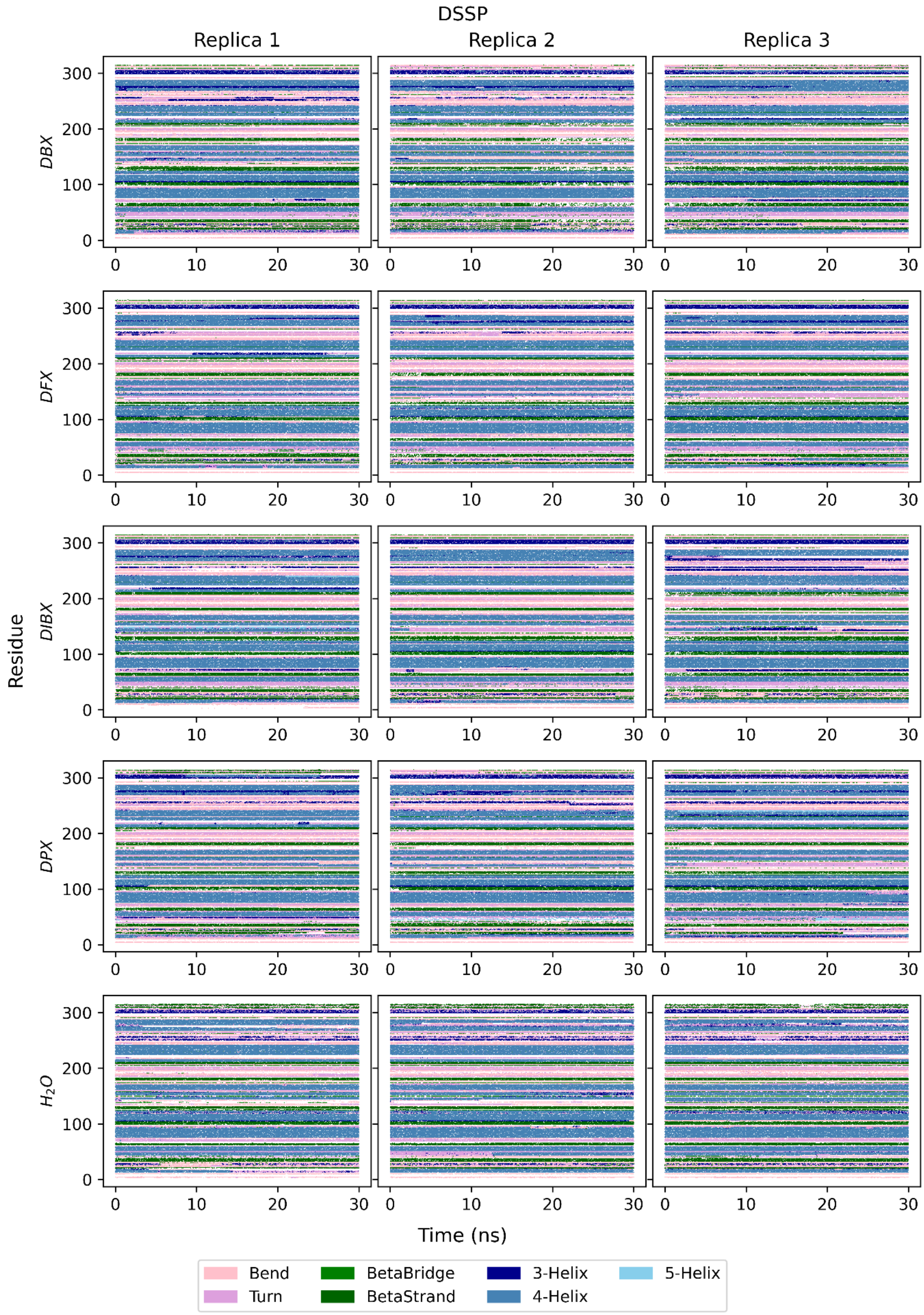

Figure S19. Secondary structure analysis (DSSP) showing the stability of the protein within the four evaluated xylose-based solvents and water.

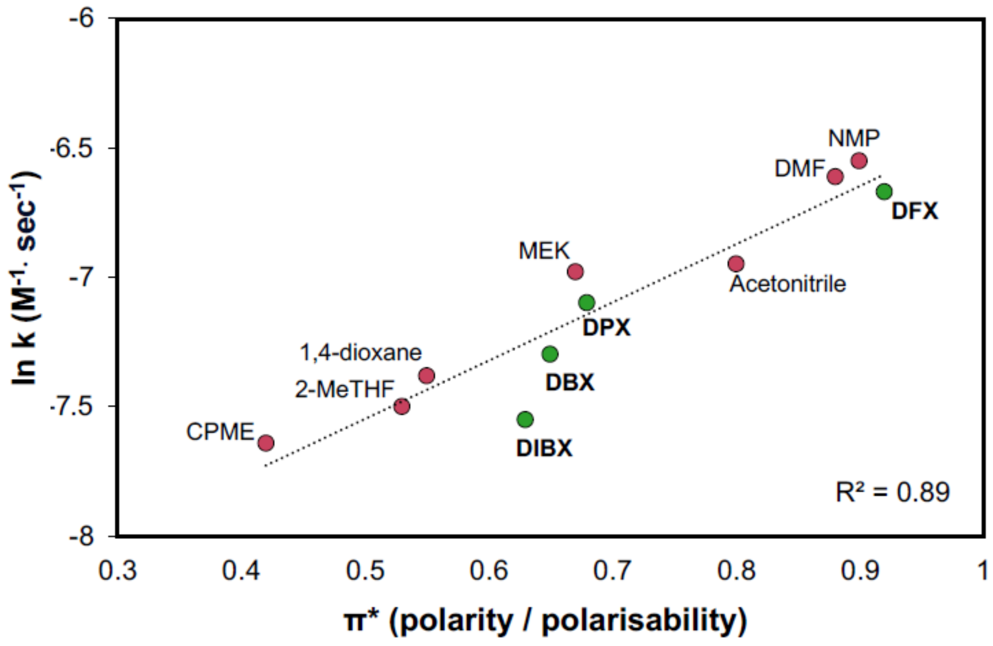

Figure S20. The relationship between solvent polarity and the natural logarithm of the rate of reaction constant for Menshutkin alkylation reaction between 1,2-dimethylimidazole and 1-bromodecane at 70 °C measured for xylose acetals in comparison to other solvents according to the procedure from our past work.[16]

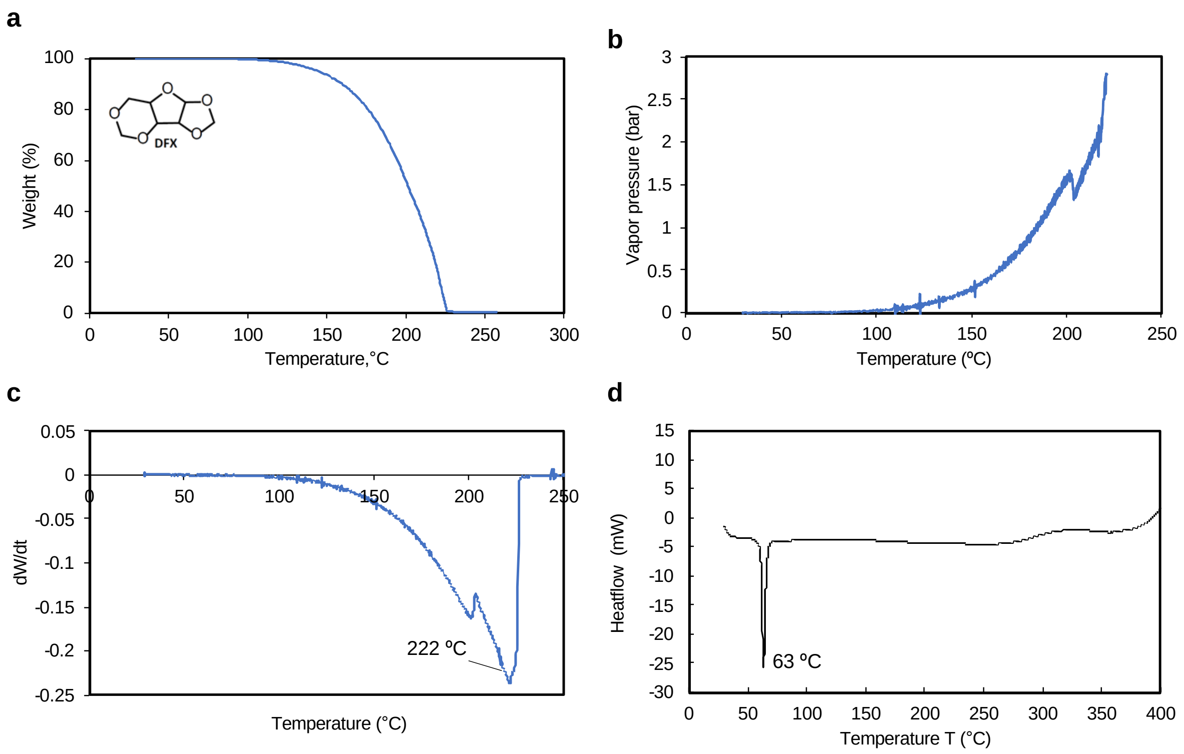

Figure S21. (a) TGA plot of DFX measured at 10 ºC/min heating rate from 25 ºC to 300 ºC. (b) Vapour pressure curve of DFX. (c) First derivative of the TGA curve. (d) DSC curve for DFX.

References

1. M. Talebi Amiri, G. R. Dick, Y. M. Questell-Santiago, J. S. Luterbacher, Nat. Protoc. 2019, 14, 921–954.
2. K. Iffland, J. Sherwood, M. Carus, A. Raschka, T. Farmer, J. Clark, “Definition, Calculation and Comparison of the ‘Biomass Utilization Efficiencies (BUE)’ of Various Bio-based Chemicals, Polymers and Fuels,” can be found under https://renewable-carbon.eu/publications/product/nova-paper-8-on-bio-based-economy-definition-calculation-and-comparison-of-the-biomass-utilization-efficiency-bue-of-various-bio-based-chemicals-polymers-and-fuels-%e2%88%92-full-version/, 2015.
3. O. H. Pardo Cuervo, C. F. Gonzalez, H. A. Rojas, J. J. Martínez, G. P. Romanelli, A. F. Peixoto, Biomass Conv. Bioref. 2023, DOI 10.1007/s13399-023-04707-7.
4. Q. Liu, Q. Liu, X. Hu, Catal. Commun. 2020, 135, 105836.
5. S. W. Fitzpatrick, Lignocellulose Degradation to Furfural and Levulinic Acid, 1990, US4897497A.
6. V. Rapinel, O. Claux, M. Abert-Vian, C. McAlinden, M. Bartier, N. Patouillard, L. Jacques, F. Chemat, Molecules 2020, 25, 3417.
7. Y. Shao, Q. Li, X. Dong, J. Wang, K. Sun, L. Zhang, S. Zhang, L. Xu, X. Yuan, X. Hu, Fuel 2021, 293, 120457.
8. Y. Liu, K. Liu, M. Zhang, K. Zhang, J. Ma, S. Xiao, Z. Wei, S. Deng, RSC Adv. 2021, 12, 602–610.
9. G. R. Court, C. H. Lawrence, W. D. Raverty, A. J. Duncan, Method for Converting Lignocellulosic Materials into Useful Chemicals, 2012, US20120111714A1.
10. J. Sherwood, M. D. Bruyn, A. Constantinou, L. Moity, C. R. McElroy, T. J. Farmer, T. Duncan, W. Raverty, A. J. Hunt, J. H. Clark, Chem. Commun. 2014, 50, 9650–9652.
11. P. Nicolet, C. Laurence, J. Chem. Soc., Perkin Trans. 2 1986, 1071–1079..
12. C. Laurence, P. Nicolet, M. Helbert, J. Chem. Soc., Perkin Trans. 2 1986, 1081–1090.
13. [1] IFRA, “IFRA ANALYTICAL METHOD DETERMINATION OF THE PEROXIDE VALUE,” can be found under https://ifrafragrance.org/docs/default-source/guidelines/20190910-revised-ifra-analytical-method-on-peroxide-value.pdf?sfvrsn=c4a931e2_0, 2019. (accessed 18 August 2023).
14. *European Pharmacopoeia, Council Of Europe : European Directorate For The Quality Of Medicines And Healthcare, Strasbourg, 2010.*
15. A. O. Komarova, Z. J. Li, M. J. Jones, O. Erni, F. Neuenschwander, J. D. Medrano-García, G. Guillén-Gosálbez, F. Maréchal, R. Marti, J. S. Luterbacher, ACS Sustain. Chem. Eng. 2024, DOI 10.1021/acssuschemeng.4c03799.
16. A. O. Komarova, G. R. Dick, J. S. Luterbacher, Green Chem. 2021, 23, 4790–4799.
17. ChemAnalyst, “Paraformaldehyde (PFA) Pricing Data, Market Analysis and Reports.,” can be found under https://www.chemanalyst.com/Pricing-data/paraformaldehyde-pfa-1195, n.d. (accessed October 20, 2024).

18. ChemAnalyst, "Butyraldehyde Pricing Data, Market Analysis and Reports.," can be found under https://www.chemanalyst.com/Pricing-data/butyraldehyde-1300, n.d. (accessed October 20, 2024).
19. C. Kohlpaintner, M. Schulte, J. Falbe, P. Lappe, J. Weber, G. D. Frey, in Ullmann's Encyclopedia of Industrial Chemistry, John Wiley & Sons, Ltd, 2013.
